# Hardware, Algorithms, and Applications of the Neuromorphic Vision Sensor: A Review

**DOI:** 10.3390/s25196208

**Published:** 2025-10-07

**Authors:** Claudio Cimarelli, Jose Andres Millan-Romera, Holger Voos, Jose Luis Sanchez-Lopez

**Affiliations:** 1 Automation and Robotics Research Group, Interdisciplinary Centre for Security, Reliability, and Trust (SnT), University of Luxembourg, 1855 Luxembourg, Luxembourg; claudio.cimarelli@alumni.uni.lu (C.C.); jose.millan@uni.lu (J.A.M.-R.); holger.voos@uni.lu (H.V.); 2 Faculty of Science, Technology, and Medicine, University of Luxembourg, 1359 Luxembourg, Luxembourg

**Keywords:** neuromorphic sensor, event cameras, event-based image processing, neuromorphic vision applications

## Abstract

Event-based (neuromorphic) cameras depart from frame-based sensing by reporting asynchronous per-pixel brightness changes. This produces sparse, low-latency data streams with extreme temporal resolution but demands new processing paradigms. In this survey, we systematically examine neuromorphic vision along three main dimensions. First, we highlight the technological evolution and distinctive hardware features of neuromorphic cameras from their inception to recent models. Second, we review image-processing algorithms developed explicitly for event-based data, covering works on feature detection, tracking, optical flow, depth and pose estimation, and object recognition. These techniques, drawn from classical computer vision and modern data-driven approaches, illustrate the breadth of applications enabled by event-based cameras. Third, we present practical application case studies demonstrating how event cameras have been successfully used across various scenarios. Distinct from prior reviews, our survey provides a broader overview by uniquely integrating hardware developments, algorithmic progressions, and real-world applications into a structured, cohesive framework. This explicitly addresses the needs of researchers entering the field or those requiring a balanced synthesis of foundational and recent advancements, without overly specializing in niche areas. Finally, we analyze the challenges limiting widespread adoption, identify research gaps compared to standard imaging techniques, and outline promising directions for future developments.

## 1. Introduction

Standard RGB cameras face considerable limitations, particularly in dynamic environments. The principle of these sensors involves capturing visual information as a sequence of frames at specific time intervals. Time-quantizing visual data at predetermined frame rates often results in temporal resolution limitations, as the frame rate is not aligned with the dynamic evolution of the scene. Consequently, significant details can be missed, especially in rapidly changing environments. Moreover, recording every pixel in each frame, regardless of changes since the last capture, leads to data redundancy, which affects the data rate and volume [1].

Instead, the limited dynamic range of standard RGB cameras often causes under- or overexposure in scenes with rapidly varying lighting conditions [2]. In addition, motion blur is another common problem in high-speed movement scenarios. Then, the latency inherent in a fixed frame rate and power consumption for processing a large amount of data, e.g., resulting from redundant information, poses an obstacle when real-time responsiveness and energy efficiency are required.

In response to these limitations, neuromorphic cameras (NCs), or event cameras, as they are named more frequently in the robotic vision research domain, represent a paradigm shift [3] in acquiring visual information compared to conventional frame-based cameras. In particular, each event camera’s pixel operates independently, i.e., with its analog circuit, continuously comparing the current brightness to a reference level [4]. When the difference exceeds a certain threshold, the pixel generates a sparse stream of event packets, e.g., the pixel’s address, timestamp, and the polarity of the brightness change, labeled with a high temporal resolution. This method allows for capturing visual information that mimics the human retina [5], approaching image acquisition with a biologically inspired process that responds more to real-world dynamics.

Unlike conventional frame-based images, event streams produced by neuromorphic cameras are fundamentally sparse and asynchronous and encode only changes in logarithmic brightness, rather than absolute intensity values. These properties introduce a range of unique challenges that require rethinking core computer vision paradigms. First, the temporal irregularity of event data, in contrast to the uniform frame rate of conventional cameras, necessitates novel representations (e.g., voxel grids and time surfaces) that preserve high temporal fidelity while supporting context aggregation. Second, the sparse and noise-prone nature of event output, driven by local contrast thresholds and sensor-level artifacts, complicates feature extraction, data association, and motion estimation, particularly in low-contrast or static scenes, limiting the reliability of tasks like SLAM and motion tracking. Third, the absence of absolute intensity makes it difficult to apply standard photometric algorithms or leverage traditional learning models. Recent methods attempt to reconstruct intensity indirectly via noise correlations or require dedicated architectures, such as spiking neural networks or asynchronous convolutional modules. These constraints and opportunities extend into the hardware domain, driving interest in event-driven processors and hybrid sensor-compute systems optimized for low-power, low-latency inference. As a result, event-based vision has driven innovations in both algorithm design, particularly for high-speed, low-power applications such as robotics, HDR imaging, and neuromorphic computing hardware itself.

Nevertheless, due to their unique capabilities, NCs are highly suitable for various applications where real-time processing, adaptability to diverse lighting conditions, and energy efficiency are critical. These include robotics, surveillance, autonomous vehicles, and other areas that require robust and efficient visual sensing. For example, NCs can provide low-latency obstacle detection, even in challenging lighting or weather conditions, which is crucial in autonomous vehicle navigation. In robotics, event cameras enable more responsive situational awareness to changes in the dynamic environment. Due to their radically different sensor modality, NCs can offer non-invasive monitoring systems, which is helpful in data-privacy-preserving scenarios like healthcare. Moreover, their low power consumption and small data volumes, i.e., sparse event packets vs. dense image frames, make them ideal for remote surveillance monitoring systems or search and rescue missions, where energy efficiency is paramount.

To date, neuromorphic vision technology and event-camera image processing have been the focus of multiple reviews, each exploring the topic from a unique perspective. These surveys encompass a wide range of topics, from sensor technology to image-processing methodologies. In Table 1 and Table 2, we provide an overview of their focus topic and highlight their key contributions. The multitude of surveys reflects the rapid development of neuromorphic vision research in recent years and the traction the field has gained, especially in the robotics and computer vision communities.

While most recent surveys tend to specialize in emerging niches or focus on specific technical components, there remains a clear need for a broader yet integrative perspective. Our work fills this gap by methodically bridging the evolution of neuromorphic vision hardware, algorithmic strategies, and real-world deployment scenarios. We aim to connect technological advancements with their practical adoption across industries by outlining the key challenges, limitations, and opportunities in this rapidly evolving field. This structured synthesis enables both newcomers and experts to grasp the interplay between sensor design, data processing, and application-driven innovation.

**Figure 1:**
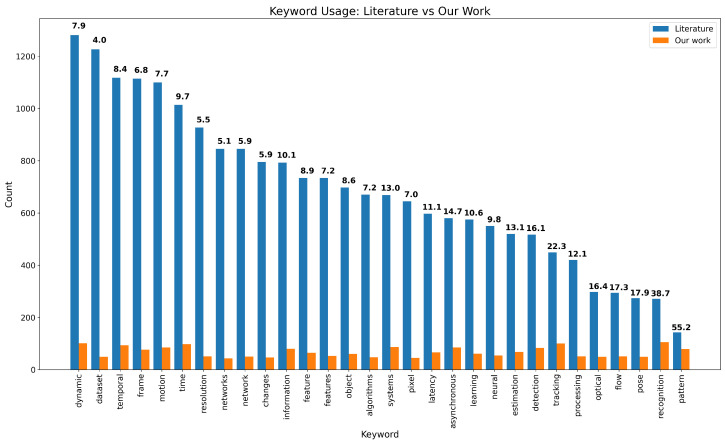
Keyword frequency analysis chart. We compare the accuracy of the keywords in the literature retrieved from https://www.dimensions.ai/ (accessed on 31 July 2025) with those used in our review. The percentage over the bars indicates the percentage covered.

To provide quantitative insight into the technological landscape of neuromorphic vision, we analyzed the keywords, titles, and abstracts from our bibliography. It consists of entries collected via Zotero and manually curated through open APIs such as arXiv, CrossRef, and SemanticScholar. Approximately 40% of the entries include abstracts. We developed a Python-based (version 3.13.2) script that parses these BibTeX-style entries, extracts the relevant fields, and performs tokenization and filtering. Common English stopwords and non-scientific terms are excluded using a predefined filter. The resulting set of keywords is further refined by applying heuristics informed by domain-specific LLM models to focus only on meaningful technical terms, finally extracting the 30 most frequent scientific keywords. Lastly, we performed a deep analysis of the research database at https://www.dimensions.ai/ (accessed on 31 July 2025) using the exact keywords coupled with “neuromorphic camera” and “event camera” strings to restrict the search to only those related to our current review scope, and we present our findings in Figure 1. In particular, the picture shows our balanced coverage on each sub-topic of the neuromorphic camera in the literature in the present review, with only two outliers in the coverage percentage for which the keyword was the least frequent in the database.

**Figure 2:**
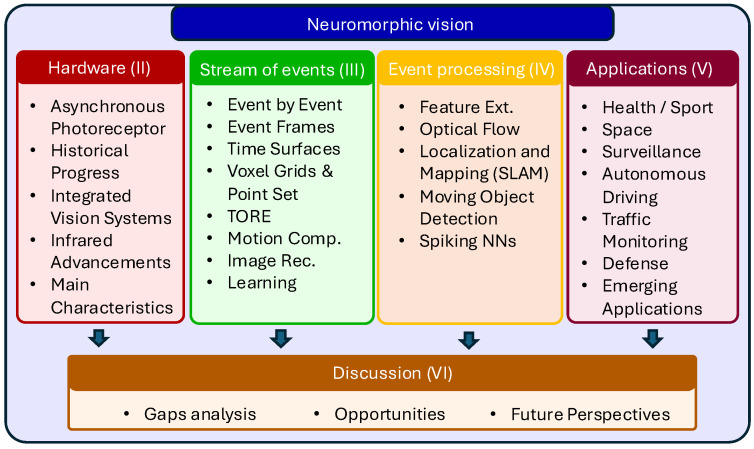
Overview of the paper’s structure, illustrating the main sections, in parentheses, and their relationships.

To guide the reader through the organization of this survey, we illustrate the paper’s structure in Figure 2. This visual abstract outlines the main thematic areas—hardware, algorithms, and applications—each contributing to the overall discussion and analysis in the final section.

In summary, with this survey, we make several distinct contributions:

- Neuromorphic Cameras’ Hardware Evolution: We present a timeline of the evolution of neuromorphic vision sensor technology, revealing the chronological progress of the hardware, how it differs from standard vision systems, and why these differences matter (Section 2).
- Event-based Image Processing and Algorithms: We examine the progression of image-processing techniques from classical methods to advanced deep learning approaches (Section 3 and Section 4).
- Application Focus: We discuss key application case studies demonstrating how the unique properties of neuromorphic cameras impact real-world solutions (Section 5).
- Gaps, Limitations, and Future Opportunities: We analyze the key challenges hindering the adoption of neuromorphic vision sensors, from hardware constraints to algorithmic gaps and real-world application barriers, while highlighting the opportunities unlocked by this radical shift in visual sensing modality (Section 6).

## 2. The Neuromorphic Vision Sensor

The initial concept of the NC invention arrived from the research group of Professor Carver Mead at Caltech with the publication of the book “*Analog VLSI and Neural Systems*” in 1989 [25]. Notably, Misha Mahowald, Mead’s student, developed during her Ph.D. from 1986 to 1992 at Caltech the first neuromorphic chip to spike events resulting from detected light intensity variations [26,27,28]. The first specialized commercial application inspired by these ideas was a motion detection system for pointing devices designed by Xavier Arreguit of CSEM for Logitech in 1996 [29].

The following discussion clarifies the pixel design and its asynchronous data output; illustrates camera models that came to the market, from visible light to the infrared spectrum; and concludes by examining the unique characteristics of event cameras that make them a distinctive technology.

### 2.1. The Neuromorphic Camera’s Asynchronous Photoreceptor

Neuromorphic cameras leverage asynchronous photoreceptors to efficiently mimic the responsiveness and energy efficiency of the human visual system, a concept that has been explored since the early developments of the silicon retina [26]. These receptors detect changes in light intensity and encode this information into discrete events, resulting in an array of pixels operating independently. This mechanism contrasts with the conventional camera’s approach of capturing entire frames regularly, thus processing and transmitting large volumes of redundant data.

Central to the operation of neuromorphic cameras is the Asynchronous Address-Event Representation (AER), an innovative communication protocol developed from the pioneering research by the Caltech group led by Carver Mead [27] and refined through subsequent research [30]. AER uses time-coded addresses to encode and dynamically transmit events between the silicon photoreceptor and the computing processor. Each event, whether an ON-event, indicating an increase in light, or an OFF-event, indicating a decrease, is defined by its pixel reference, timestamp, and polarity.

**Figure 3:**
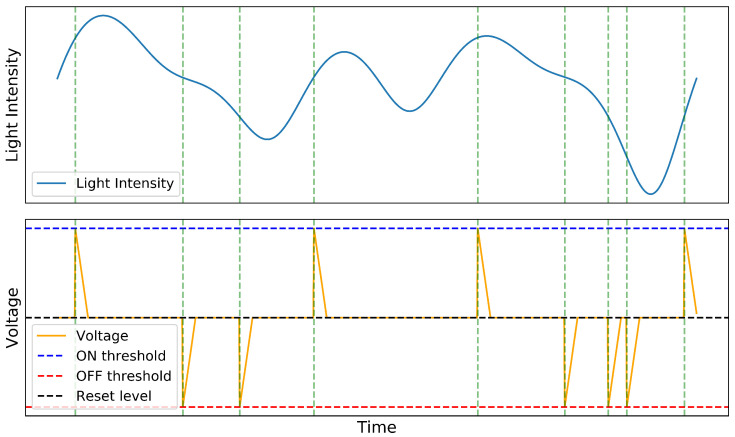
The asynchronous output of an operating event-camera photodiode. Inspired from an image in [4]. Dashed green lines indicate when a change exceeding the voltage threshold encoded in the photoreceptor is detected.

The neuromorphic photoreceptors respond to light intensity variations on a logarithmic scale. This capability allows the sensor to handle various lighting conditions effectively, from dim to bright. Each pixel analog circuit, as the primary designed in [31], detects changes that surpass a voltage threshold encoded in the photoreceptor, triggering the transmission of the AE packet, as illustrated in Figure 3.

As explored by Steffen et al. [22], this sophisticated protocol incorporates a digital bus system and multiplexing strategies that allow all pixels to transmit their information over the same line efficiently and asynchronously, significantly reducing power consumption and data volume.

Further refining the process, the AER’s implementation via address encoders generates unique binary addresses for each pixel event. This overall strategy highlights the role of AER in transmitting only essential visual information while discarding irrelevant static scenes and ensuring effective responses to rapid changes in the environment [32].

### 2.2. Progress in Visible-Light Event-Camera Models

**Figure 4:**
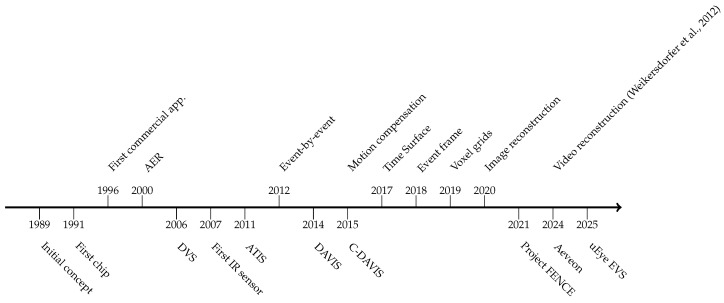
Timeline of pivotal milestones in developing the neuromorphic vision sensor. Events related to software developments are depicted above the axis, whereas hardware-related milestones are positioned below it [33].

With the theoretical progress made in the 1990s, several research organizations started producing the first neuromorphic sensors. Toby Delbruck proposed the first generic event camera in 2008 in collaboration with Patrick Lichtsteiner and Christoph Posch under the dynamic vision sensor (DVS), the earliest event-camera technology. Lichtsteiner, Posch, and Delbruck [4,31] proposed a novel silicon retina design that outputs AER in a 128 × 128 pixel grid. Since the DVS’s inception, many companies have commercialized VGA to megapixel resolution event cameras, from a small reality like CelePixel or Insightness to a technological giant like Samsung, producing further innovations of the original sensor. Event-camera expression gradually took place in the last few years to highlight the AER output of neuromorphic vision devices and differentiate them from their standard camera counterparts. A summary of key hardware and software advancements in neuromorphic vision is illustrated in the timeline shown in Figure 4.

In 2014, Christoph Posch co-founded Chronocam (now Prophesee) in France, focusing on the development and commercialization of Asynchronous Time-based Image Sensor (ATIS) technology, outlined in Posch’s 2011 research at the Austrian Institute of Technology [34]. The ATIS marks a significant advancement in event-camera technology, merging the temporal contrast-detection capabilities of the DVS with innovative time-based intensity measurement pixels. This integration allows the ATIS to capture event-based data and provide absolute brightness measurements with high accuracy. However, incorporating a pulse-width-modulated (PWM) intensity readout mechanism for each DVS pixel, aimed at enhancing reconstruction and recognition capabilities, necessitated an extra photodiode per pixel, effectively doubling the pixel size. Moreover, because the PWM readout process required the transfer of triple the data amount, the ATIS latency is significantly increased, particularly impacting the sensor’s ability to capture fast-moving or dimly lit objects. Despite this, the sensor’s QVGA resolution (e.g., 304 × 240) substantially improves detail and image quality over the original DVS. Furthermore, the ATIS addresses critical limitations of traditional imaging systems by significantly reducing temporal redundancy and delivering a high dynamic range (143 dB static and 125 dB at 30 FPS). Further developments of ATIS technology have led to multiple sensor generations, such as Prophesee Metavision GEN3, including collaboration with Sony on the IMX636 and IMX637 sensors. These sensors, featuring stacked CMOS technology, underscore the ATIS’s ongoing evolution and potential in various high-performance imaging applications.

The Dynamic and Active-pixel Vision Sensor (DAVIS) [35], developed by IniVation, represents a significant advancement in vision sensor technology. Unlike its predecessors, the DAVIS integrates neuromorphic event-driven and active-pixel sensor (APS) functionality within the same photodiode. This innovative design enables the DAVIS to interleave event data with conventional intensity frames, using a shared pixel to generate grayscale and event data. The pixel architecture of the DAVIS offers several benefits: it achieves a dynamic range of 130 dB for event detection and 51 dB for grayscale intensity frames. Additionally, it features a minimized latency of just 3 μs. Despite its dual functionality, the pixel area in the DAVIS is only marginally larger (about 5%) than that of a standard DVS pixel, resulting in a slightly reduced high dynamic range compared to the ATIS but in a more compact form factor.

The Color Dynamic and Active-Pixel Vision Sensor (C-DAVIS) represents a significant advancement, building upon the foundations of the earlier DAVIS model [36]. This sensor combines monochrome event-based pixels with a five-transistor APS architecture integrated under a Red, Green, Blue, and White (RGBW) color filter array. Capable of outputting both rolling or global shutter RGBW-coded VGA resolution frames and asynchronous monochrome QVGA temporal contrast events, the C-DAVIS excels in capturing vibrant color details as well as tracking swift movements with remarkable temporal precision. This blend of capabilities is efficiently packed into a compact design, featuring a 2 × 2-pixel RGBW unit with dimensions of merely 20 μm × 20 μm, showcasing the C-DAVIS’s ability to combine high-resolution color imaging with fast, event-based motion detection.

In 2023, IniVation introduced the Aeveon sensor, an advancement in neuromorphic vision technologies, to address the limitations of previous models like the DAVIS. The Aeveon is designed to allow each pixel to generate several event types, including full pixel value (RGB), multi-bit and single-bit change events, and area events. Moreover, it employs a stacked sensor design with Adaptive Event Cores, merging characteristics of neuromorphic sensors with frame-based sensors. This design is compatible with various pixel types, from standard RGB to infrared. Furthermore, the sensor offers the flexibility to select an adaptable region of interest (ROI), where the user can focus the event stream reception, similar to an attention mechanism. With its unified solution, the Aeveon should facilitate integration with existing systems while providing an immediate replacement for the current vision modules and a pathway to introduce new event-based features gradually.

In Table 3, we provide a list of currently commercialized event cameras. Other sensors have been described in the literature, such as in [3,19]. However, not all are available for purchase, e.g., the early DVS128 and DVS240 from IniVation, or are not easy to procure online, such as the models produced by Insightness, Samsung [37], and CelePixel [38,39]. Recently, IDS and Prophesee partnered to create the new uEye EVS camera series [40], enabling ultra-fast imaging (sub-100 μs resolution) and significantly reducing data processing and power consumption.

Notably, the price of such devices is still a few thousand dollars, making them currently functional only for industrial purposes. The cost of production is the main obstacle to the diffusion of event cameras in larger commercial markets until mass production of the sensor’s silicon. However, recent partnerships, such as the announced collaboration between Prophesee and Qualcomm, allow us to foresee that event cameras may soon be adopted for mobile platform imaging. As an example of this trend, Google is adding event-based vision to its Visual Intelligence and Android XR platforms—paving the way for advanced AR glasses [41,42]. As adoption expands and commercial partnerships grow, the focus is increasingly on the technical challenges that remain, motivating further innovation in sensor integration and system design.

### 2.3. Toward Fully Integrated Neuromorphic Vision Systems

Recent progress has not only advanced pixel architectures, system integration, and commercial availability but also highlighted several remaining technical bottlenecks. Many visible-light event cameras, while achieving improved resolution, dynamic range, and frame fusion capabilities, continue to rely heavily on external or off-chip processing, which can limit their potential in latency-critical, power-constrained, or edge-driven applications. As the field matures, an emerging line of research shifts from simply refining sensing hardware to fundamentally rethinking the coupling of sensing and computation, aiming to achieve real-time, ultra-efficient, and adaptive vision in complex environments. The latest examples of this paradigm are seen in neuromorphic architectures that tightly merge event-driven sensing and in-sensor computation, enabling a leap beyond the traditional boundaries of event-camera design.

In addition to recent advances in pixel design and readout circuits, a new wave of neuromorphic sensors is fundamentally redefining the event-camera landscape by tightly merging sensing and computation at the hardware level. For example, the Speck chip [43] exemplifies a fully integrated neuromorphic sensing and computing system on a chip, which unites a dynamic vision sensor with a fully asynchronous event-driven spike neural network processor. This architecture enables truly “always-on” operation where the computation is activated only by incoming events, pushing idle power consumption to below one milliwatt. As a result, Speck delivers robust, real-time, ultra-low-power processing for high-speed event streams, opening up practical opportunities for edge intelligence in severely power-constrained scenarios.

Complementing this approach, the Tianmouc chip [44] brings a dual-path design inspired by biological science that mimics the parallel ventral (cognitive) and dorsal (action) streams of the primate visual system. Using a hybrid pixel array and parallel, heterogeneous readout chains, Tianmouc achieves high-resolution, high-precision perception and simultaneously delivers rapid, sparse, event-based responses in highly dynamic and unpredictable scenes. Notable system capabilities include operation at up to 10,000 frames per second, a dynamic range reaching 130 dB, and considerable bandwidth reduction through sparse event encoding. This complementary vision paradigm, validated on demanding open-world tasks such as autonomous driving, underlines the importance of accurate scene understanding and rapid recognition of rare and safety-critical events.

Together, these integrated sensing–computing systems exemplify a clear trend of moving beyond conventional event-camera designs. This means switching to a highly adaptive, energy-efficient, and genuinely edge-capable neuromorphic vision platform, where sensing and visual perception reasoning are seamlessly fused at the hardware level.

### 2.4. Development of the Infrared Neuromorphic Vision Sensor

In the field of neuromorphic chip research, the primary emphasis has been on the visible waveband. However, there are certain situations when objects of interest become challenging to perceive due to fluctuations in scene illumination. This problem becomes more pronounced when the photons of interest are not emitted in the visible waveband, such as during nighttime or when the atmospheric conditions are unsuitable for the visible waveband.

To this aim, one approach is to shift or extend the measurable light spectrum toward the infrared region [45]. These sensors are typically categorized based on the wavelength range they are sensitive to, which includes short-wave infrared (SWIR), mid-wave infrared (MWIR), and long-wave infrared (LWIR). Each sensor type has its advantages and is suitable for different applications. SWIR cameras typically operate in the wavelength range of 1 μm to 2.5 μm. SWIR sensors can distinguish between organic and inorganic materials, making them ideal for various applications, including recycling, the food industry, drones in agriculture to detect water shortages, military applications involving lasers, and improving atmospheric transmission. MWIR cameras usually use a wavelength range of 3 to 5 μm. MWIR sensors are known for their ability to detect thermal radiation emitted by objects at high temperatures, making them suitable for airborne and ground-based surveillance, thermography, and gas detection applications. LWIR cameras typically operate in the wavelength range of 8 to 14 μm, commonly referred to as the thermal imaging region, as it allows the detection of the thermal radiation emitted by objects or materials at ambient temperature. LWIR sensors suit thermal imaging, night vision, and medical diagnostics applications. See Table 4 for a comparison of infrared neuromorphic cameras by wavelength.

Posch et al. [46] developed the first IR event-based sensor by coupling a microbolometer array with typical DVS readout circuitry [47]. A microbolometer is a thermal sensor that detects thermal infrared radiation based on the variation in its temperature-dependent electrical resistance, and it is sensitive in the LWIR range. It can be integrated with complementary metal–oxide–semiconductor (CMOS) readout circuitry. However, the time constant of current microbolometers (around 10 ms) is relatively slow and does not allow us to take full advantage of the NC technology. Alternative IR technologies, such as cryogenic IR quantum sensors for MWIR and LWIR or InGaAs for the SWIR region, seem more promising.

Furthermore, SCD is announcing the preparation of a product in SWIR [48]. This product, with a resolution of VGA (15 μm pitch) for the imaging mode and quarter-VGA for event-based output, should be available shortly under the name SWIFT-EI. In addition, DARPA has launched the FENCE project [49] to develop event-based infrared cameras sensitive to the infrared band above 3 μm, supposedly including MWIR and LWIR.

### 2.5. Main Characteristics of Event Cameras

The unique design and operational characteristics of neuromorphic vision sensors offer several advantages over traditional vision technologies. Here, we list the most remarkable:

- **High temporal resolution**: NCs can capture fast-moving objects and obtain greater detail of the evolution of the motion without having to interpolate between frames. Light intensity change is detected by analog circuits with high-speed response. Then, a digital readout with a 1MHz clock timestamps the event with microsecond resolution [3].
- **Low latency**: Event cameras have low latency, meaning they can respond quickly to environmental changes. Contrary to the traditional camera, NCs do not have a shutter, so there is no exposure time to wait for before transmitting brightness change events. Therefore, latency is often tens to hundreds of microseconds under laboratory conditions to a few milliseconds under real conditions [3].
- **High dynamic range**: NCs can capture bright and dark scenes without losing detail. This property is particularly beneficial for sudden changes in illumination that can cause overexposure or in low-light environments where the scene may appear too dark. This property is due to the logarithmic response at the photoreceptors. Hence, whereas static vision sensors have a dynamic range limited to 60 dB because all the pixels share the same measurement integration time (dictated by the shutter), event cameras can go over 120 dB for their independent pixel operations.
- **Low power**: NCs consume much less energy than traditional cameras, making them suitable for low-power devices and applications. Notably, all pixels are activated independently based on the illumination changes each one detects, and the analog circuit is very efficient. As a result, the NC’s power demand can go as low as a few milliwatts.
- **Sparsity**: NCs provide data only when there is a change in the scene. Hence, the amount of data that needs to be processed is reduced. As a result, the event cameras may output up to 100× less data than traditional cameras with similar resolution. To fully exploit this technology, NCs must be coupled with chips capable of processing the events with algorithms such as spiking neural networks designed to maintain the low-power premises and keep events’ intrinsic asynchronous nature intact. SynSense is one such company that develops neuromorphic chips, such as the DYNAP-CNN chip, for ultra-low-power applications, e.g., IoT devices, and can be integrated with the same chip with the DVS pixel array, as demonstrated by IniVation Speck.

## 3. Working with Stream of Events

**Figure 5:**
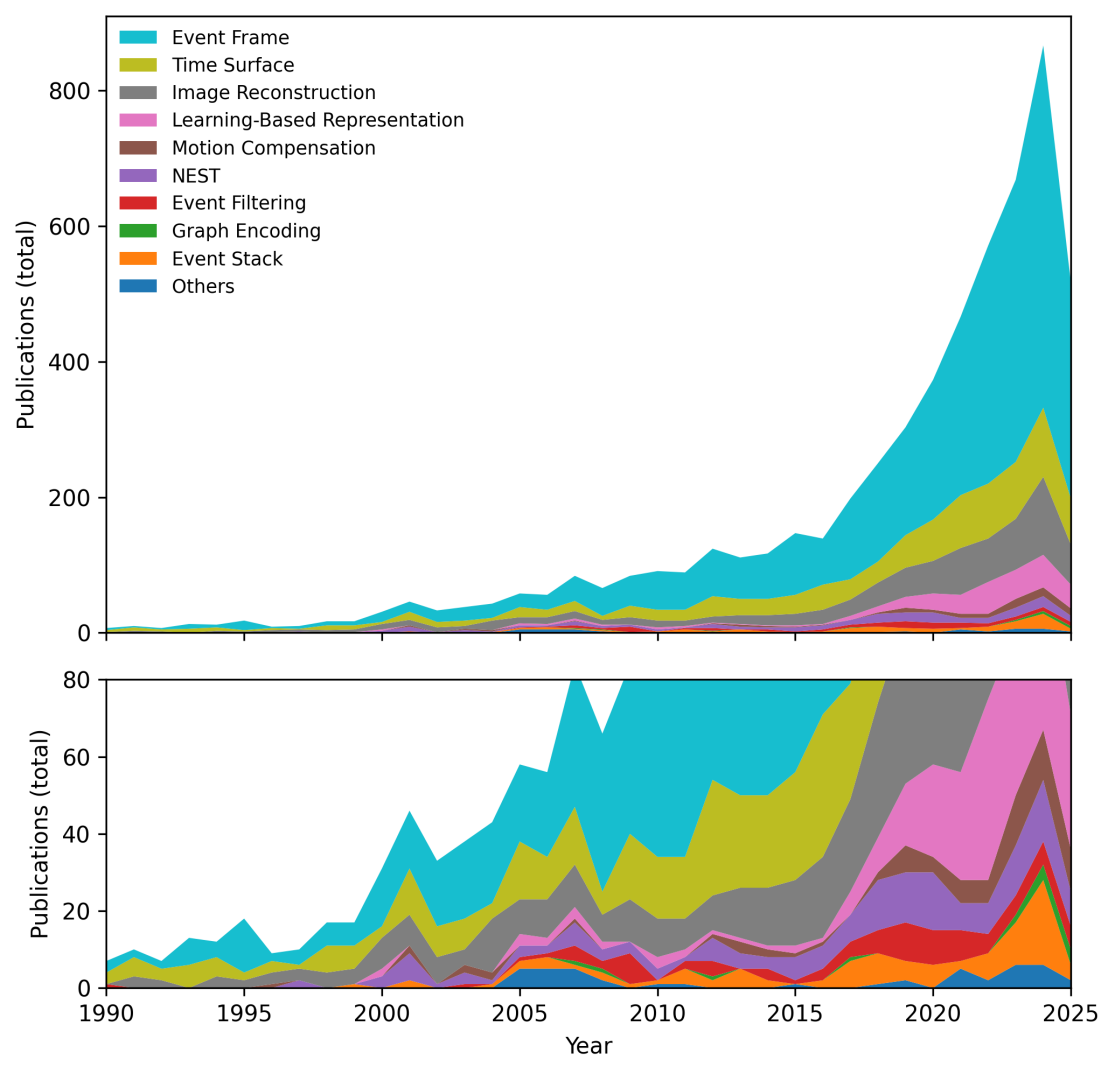
Event representation trends over time. The figure’s legend entries are ordered by descending number of publications in 2025. “Others” includes voxel grid, Spike Tensor, TORE volume, and Cloud Encoding.

In this section, we explore various representations of the event stream and how they are processed for specific applications. Figure 5 illustrates the trend in event representation methods over time, based on a search with target queries in the Dimensions.ai database, highlighting the increasing popularity and development of these methods. This chart provides a quantitative view of the evolution of event representations, as discussed in the subsections of this section.

The intrinsically different nature of the neuromorphic sensor compared to traditional cameras necessitates new approaches to represent the information captured and to process it in a format suitable for input into specific image-processing algorithms. Neuromorphic cameras (NCs), also known as event cameras, trigger asynchronous events for those pixels that detect a brightness change exceeding a certain threshold—caused either by camera motion or by moving objects in the scene. As a result, the sensor outputs so-called events that encode not only the spatial location of the pixel (*x* and *y* coordinates) but also the polarity of the brightness change (positive or negative) and a precise timestamp.

**Figure 6:**
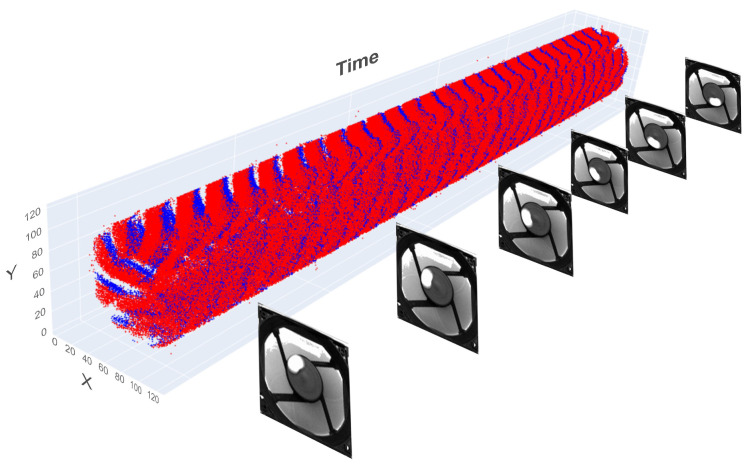
The stream of events as blue points interleaved with grayscale image frames at a fixed rate. While ON events are represented in red, OFF events are represented in blue. Image credits to [50].

An example of such an event stream is shown in Figure 6, captured alongside fixed-rate grayscale frames. This visual juxtaposition highlights the drastic difference from the classic image input format.

This fundamentally different data format calls for new analysis methods. One direction is to develop algorithms that operate directly on the sparse and asynchronous event stream. Alternatively, the event stream can be converted into more conventional representations, which require fewer or no modifications to existing algorithms. Another design choice is whether to process individual events to minimize latency or to group events into packets, which can then be transformed into other formats and offer more contextual information. In either case, prior context must be considered, as a single event alone lacks sufficient information [51].

To translate the distinctiveness of each preprocessing strategy to explicitly grouped families, Table 5 contrasts them by *what they preserve vs. discard*, typical tasks, and core limitations. Methodologically, we normalize each representation family along five axes: (i) the family itself, (ii) which signal properties are *preserved* (temporal precision, polarity, sparsity, local spatial context), (iii) which are *lost* (e.g., microsecond timing, long-term history), (iv) typical downstream tasks, and (v) core limitations. This axis set is distilled from recent surveys (e.g., [9,15,16]) and the present review, so that readers can understand which preprocessing is more appropriate for the use case and navigate the approaches, even though they might have been treated with slightly different naming by different authors.

### 3.1. Event-by-Event Processing

Among the methods that process event-by-event, we recognize probabilistic Bayesian filters, e.g., Kalman filters (KFs) or particle filters (PFs), and spiking neural networks (SNN). Bayesian filtering is a statistical method that relies on Bayesian theory to maintain a probability distribution over the possible states and update this distribution as new data becomes available. Examples of this approach are found especially concerning pose estimation [33,68]. Gallego et al. [69] demonstrate the tracking of the 6-DoF pose of a DVS camera from an existing photometric depth map. Kim et al. [70] provide accurate rotational motion tracking while reconstructing high dynamic range spherical mosaic views from gradient images using Poisson solvers [71]. Scheerlinck et al. [72] introduced a continuous-time formulation for intensity estimation and fusion of events with image frames using a complementary filter. Additionally, the paper provides a new dataset for evaluating image reconstruction. Later, they proposed a method to compute the spatial convolution of a linear kernel with the output of an event camera, using an internal state that encodes the convolved image information and demonstrates the application of the proposed method to Harris corner detection [73].

Instead, the spiking neural network (SNN) [52] is a type of neural network that models the behavior of biological neurons and the communication between them using discrete spike signals. Hence, they can process asynchronous inputs by encoding the spike’s timing. Typical use of these networks involves character recognition, as demonstrated in [74], where an SNN architecture named HFirst exploits the event’s temporal information, integrating Integrate-and-Fire neurons with a Winner-Take-All selection strategy. While in HFirst, the network comprises handcrafted kernels such as the Gabor filter [75], the SLAYER algorithm [76] demonstrates how to handle the non-differentiable nature of the spike signal. It performs a modified backpropagation to learn the weights and axonal delay parameters of SNNs. The SNN’s asynchronous and sparse spiking pattern can be exploited by specific neuromorphic hardware, such as the Intel Loihi [77], to achieve highly power-efficient models compared to the traditional Deep Neural Networks (DNNs) running on GPUs. Unfortunately, this type of hardware is not yet commercially available, so we have to rely on conventional chips, on which SNNs do not have the same energy efficiency properties. Therefore, converting packets of events into 2D or 3D representations is often convenient to process by computer vision algorithms that can better use the currently available hardware resources. In the following, we will explore the most common ways to preprocess event packets and transform them into a format that allows standard image-processing algorithms to analyze them.

### 3.2. Event Frame

One of the first 2D representations of event packets is the event frame, which helps process event streams using traditional computer vision techniques, algorithms, and tools not explicitly designed for event-based data. Furthermore, the event frame representation can be used to visualize the events in a way familiar to human observers. In this representation, the events accumulate over time and are used to update a brightness increment image. The advantage of event frames is that their frame rate can be adapted to the use case. However, they have severe limitations compared to other representations, such as time surfaces or voxel grids, in capturing the temporal dynamics of the events. Examples of event frame applications include optical flow [54], stereo vision [78], and deep learning applied to steering angle prediction [55].

### 3.3. Temporal Binary Representation

A particularly compact and efficient event aggregation strategy is the Temporal Binary Representation (TBR) [53]. TBR first stacks short-interval binary event frames (presence or absence of an event per pixel per time slice) and then losslessly encodes these as decimal values via binary-to-decimal conversion. This enables preserving fine-grained temporal information over a longer window in a single, compact frame while allowing standard CNNs to process the result. While TBR is highly effective for gesture/activity/action recognition with low memory and computation, the loss of precise event count and timing can limit its suitability for tasks such as optical flow or fine time-resolved regression.

### 3.4. Time Surface and Surface of Active Events (TS/SAE/SITS)

Another popular representation is the time surface (TS) [56,57]. A TS is a spatiotemporal representation of an event and its surrounding activities that uses the arrival time of events from nearby pixels. It is a 2D array where each pixel stores the time of the most recent event at that location, with the pixel’s intensity indicating the event’s time. Time surfaces are a time-resolved version of an image and can be used to analyze the dynamics of an event stream over time. Recent events are emphasized over past events using an exponential kernel, and normalization is used to achieve invariance to motion speed. Each pixel value can be computed by filtering events within a space–time window to reduce sensitivity to noise. However, time surfaces compress information by keeping only one timestamp per pixel, which can reduce their effectiveness in scenes with frequent events or textures.

Surface of Active Events (SAE) [79] is similar to the time surface but typically focuses on the active regions within the event stream. While the time surface is a direct encoding of the most recent event per pixel, SAE can highlight areas of activity and capture faster dynamic events more effectively. This makes it particularly suited for tasks like corner detection and visual SLAM, where distinguishing rapid changes is crucial.

In addition to these, the Speed-Invariant Time Surface (SITS) [58] is an extension of the time surface that addresses the limitation of sensitivity to object speed in typical TS representations. SITS introduces a novel formulation that makes the time surface invariant to object speed, which is crucial for corner detection and motion tracking, especially under fast and abrupt motion. This approach allows the surface to remain robust even when the direction of motion changes rapidly, a typical challenge for event-based cameras in real-world conditions. Thus, while TS and SAE capture the basic temporal and spatial relationships in event data, SITS adds an extra layer of speed invariance, offering enhanced stability and performance for dynamic and fast-moving scenarios, such as corner detection and real-time motion tracking.

### 3.5. Voxel Grids and Point Sets

Voxel grids involve dividing a 3D space into a regular grid of voxels, essentially 3D pixels, associated with a value representing the features or characteristics of the object or scene at that location. For example, each voxel contains the number of events within a spatiotemporal volume in the event-camera context. The temporal dimension is discretized in multiple bins, and the voxel value is found by bilinear interpolation [59]. Voxel grids help represent volumetric data in a structured way that neural networks can process efficiently.

A similar approach represents events directly as 3D point sets, where each point is associated with an event fired at a particular time. For example, Benosman et al. [79] employ this representation to estimate motion velocity as a vector proportional to the slope of a plane fitted on the set of points.

### 3.6. TORE Volumes

Event cameras generate asynchronous, sparse event data that must be transformed into a format usable by conventional computer vision algorithms. Traditional representations, such as voxel grids, often suffer from information loss due to temporal binning. The *Time-Ordered Recent Event (TORE) volumes* [62] aim to address this limitation by retaining the *K* most recent events (timestamps and polarity) for each pixel, thereby maintaining the high temporal fidelity of the original event stream. This bio-inspired approach utilizes a compact 4D buffer that efficiently stores recent events without resorting to fixed temporal windows. TORE volumes preserve the sparse nature of event data, making them computationally efficient and suitable for GPU processing. They have demonstrated significant improvements across a range of tasks, including denoising, event-based image reconstruction, and object classification, and have become a promising alternative to discretized voxel stacks in deep learning pipelines.

### 3.7. Graph/Event-Cloud Encodings

Traditional event-processing approaches often convert sparse event data into dense-grid-like representations, which can lose valuable temporal and spatial precision. Instead, graph/event-cloud representations aim to preserve this sparsity and fine-grained temporal resolution by treating each event (or group of events) as a node in a dynamic graph. In this approach, events are represented as nodes with attributes such as spatial coordinates, temporal information, and polarity, and edges are established between neighboring nodes based on their spatiotemporal relationships. This enables the use of Graph Neural Networks (GNNs) to operate directly on asynchronous, sparse data, significantly reducing computational costs by only updating relevant parts of the graph as new events arrive [63,64]. This method aligns event data with established point-cloud networks, preserving both sparsity and precise timing, two key advantages of event-based vision systems. However, the trade-off lies in the added graph construction overhead and the need for techniques like fixed-size sampling or clustering to enable efficient batch processing during training.

### 3.8. Motion Compensation

Motion compensation [69] is a technique that represents events as image frames to reduce the motion blur and visualize sharp edges. It involves accumulating events over a certain period and using them to update an image representation of the scene that considers the motion. The intuition is that event cameras capture how edges move in the scene and are used to align the events that trigger them. Hence, we optimize an objective function called the focus function [80] to find the trajectories that warp the event back to a reference time to maximize the visual sharpness. As a result, the resulting image is sharper, making it more informative and interpretable than a raw event stream. Hence, the feature extraction [81] and visual odometry [82] tasks are easier to approach using the produced sharp-edge map. In addition, motion compensation can be used with other event representations, such as time surfaces [83] or 3D point sets [84].

### 3.9. Image Reconstruction

Image reconstruction involves obtaining grayscale frames of the scene from accumulated events. If standard camera images are available, they can be fused to add more information and overcome visual defects such as motion blur and limited dynamic range. Some of the most relevant image reconstruction techniques for these types of cameras include the following:

- **Spike-based image reconstruction** [85] involves accumulating spike data over time and reconstructing an image of the scene. One common approach is to use a spike-based reconstruction algorithm that considers the spatial and temporal patterns of the spikes to recreate a picture that closely approximates the original scene.
- **Adaptive filtering** [86] filters out noise and artifacts in data captured by event cameras. Because these cameras capture data asynchronously and at high temporal resolution, there is often a lot of noise in the data that can interfere with image reconstruction. Adaptive filtering techniques use a combination of statistical analysis and machine learning algorithms to filter out the noise and improve the quality of the reconstructed image.
- **Compressed sensing** [87] can reconstruct high-quality images from a relatively small quantity of data using a combination of algorithms and mathematical models.
- **Deep learning** [88] methods entail using neural networks to learn patterns in visual data and generate high-quality reconstructed images. This technique involves training a neural network on a large dataset of visual data and using it to reconstruct images from the sparse data captured by NCs and event cameras. Deep learning has shown promising results in improving the quality of reconstructed images from these types of cameras.

### 3.10. Learning-Based Representations

Learning-based approaches have emerged to address the limitations of handcrafted event stream representations. Rather than relying on fixed grids or time surfaces, these methods explicitly learn representations that are tailored to the target task, maximizing the retention of relevant visual or temporal information. For example, Event Spike Tensors (ESTs) [61], Matrix-LSTM [60], and Neural Event Stacks (NEST) [65] are all end-to-end architectures that learn optimal spatiotemporal features for classification or reconstruction. In particular, Teng et al. [65] propose Neural Event Stacks (NEST), a novel spatiotemporal encoding that respects physical constraints while effectively capturing motion dynamics. Their learned representation achieves state-of-the-art performance in image enhancement tasks such as deblurring and super-resolution.

Annamalai et al. [89] introduce a deep learning memory surface, which encodes temporal motion history directly from sparse events. Designed for anomaly detection, this representation preserves the asynchronous nature of the data while enabling efficient spatiotemporal analysis. Building on this, Vemprala et al. [90] use event variational autoencoders (VAEs) to handle environmental changes effectively. Schaefer et al. [64] process event data as evolving spatiotemporal graphs, named AEGNN. Unlike previous methods that convert event streams into dense representations, AEGNN treats events as sparse data, updating only relevant parts of the graph.

Guo et al. [91] address the efficient representation of volumetric videos with feature grids and introduce dynamic codebooks for storage optimization. Wang et al. [92] develop an adaptive sampling approach that dynamically selects the most relevant events in the input stream. They also introduce EAS-SNN, a spiking neural network (SNN), to enhance temporal learning by using recurrent connections that preserve context over time. Gu et al. [67] improve event-based video reconstruction by learning contrast-threshold-adaptive parameter representations, addressing issues like blurry outputs and artifacts.

Furthermore, new metrics such as the Gromov–Wasserstein discrepancy (GWD) [66] have been proposed to efficiently select among handcrafted representations based on how well they preserve task-relevant information, without requiring expensive retraining. These learning-based and metric-driven approaches enable fine-grained management of what information is preserved or discarded, at the cost of increased computational complexity and a reliance on sufficient annotated training data.

## 4. Event Stream Processing Algorithms

**Figure 7:**
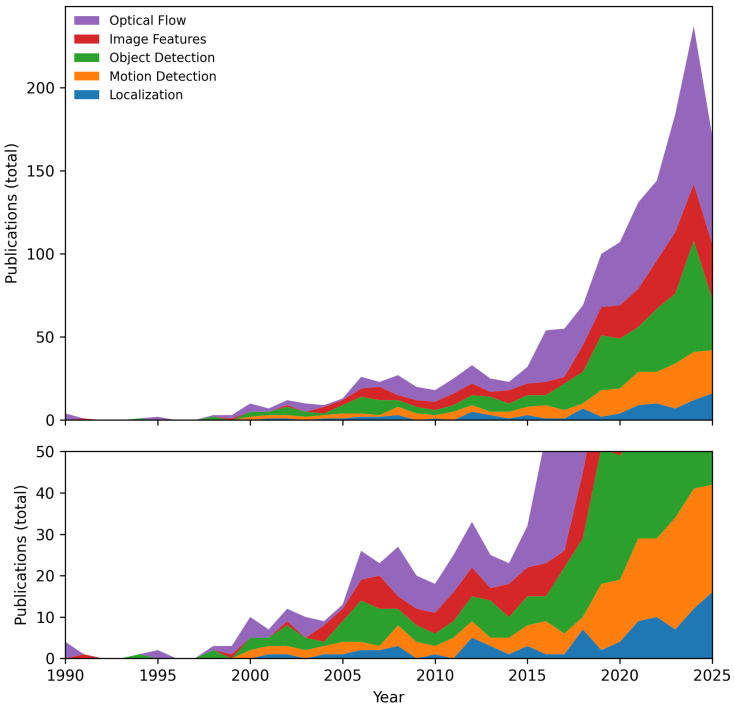
Processing algorithm trends over time. The figure’s legend entries are ordered by descending number of publications in 2025.

In this section, we delve into the algorithms used for processing event streams, focusing on their capabilities, challenges, and applications. Figure 7 illustrates the trends in event stream processing algorithms over time, based on a search with targeted queries in the *Dimensions.ai* database. This chart provides insight into how the research landscape has shifted towards more efficient and specialized processing algorithms.

### 4.1. Extraction and Tracking of Image Features

Identifying distinctive and informative features in visual data is the first step to further analyzing and understanding the surrounding world through the eyes of the camera. Therefore, extracting significant features is critical to distill sensorial input into condensed information that more complex algorithms can use. In practice, features enable visual tasks for a higher level of comprehension or situational awareness [93], like object recognition, image retrieval, camera localization, or 3D reconstruction. In traditional image analysis, features are extracted from pixel intensity patterns corresponding to geometric structures like corners and edges. Classical methods such as Harris [94], HOG [95], FAST [96], SIFT [97], SURF [98], and ORB [99] enable feature detection and description, ensuring robustness to transformations in scale, rotation, and illumination. These methods rely on dense image frames, where feature vectors encode the appearance of local pixel neighborhoods for matching and tracking.

In contrast, event-based vision requires adapting feature extraction methodologies due to its sparse and asynchronous nature. This necessitates feature detection techniques that leverage the temporal structure of event streams rather than relying on fixed-frame representations. Vasco et al. [100] propose an adaptation of Harris, while [101] advanced a version of the FAST corner detection developed to work on time-surface representations of event streams. Instead, Clady et al. [102] find corners as the intersection of planes fitted on the time surface. Alzugaray and Chli [103] presented an efficient version of eFAST for asynchronous corner detection called Arc. Subsequently, they built the ACE tracker [104] that uses a normalized local region descriptor applied to corners. FA-Harris [105] is a faster corner detection method inspired by the Harris detector. To achieve speed, they introduce a Global Surface of Active Events (G-SAE) unit and corner candidate selection and determine detection scores, showing improved accuracy performance. Li et al. [106] move towards more complex descriptors constructed using the gradient information from Speed-Invariant Time Surfaces (SITS) [58]. DART [107] uses a log-polar grid to obtain a robust descriptor valuable for object detection and tracking. Recently, deep learning-based descriptors have started to appear. Huang et al. [108] propose a variation of the TS representation, Tencode, that considers polarities and creates a multitemporal resolution input for training a deep network inspired by the Superpoint architecture [109]. Their approach, EventPoint, shows promising results concerning previous methods, e.g., [110], where Harris corners are extracted from predicted image gradients instead.

Extraction and tracking are intertwined tasks, as features are good if we can track them for long frame sequences [111]. Feature tracking refers to establishing correspondences or matches between visual features over time in a sequence of images or video frames through a process usually referred to as data association. Feature tracking is typically performed by detecting the key points in the first frame of the sequence and then matching them with key points in subsequent frames. Matching can be achieved using various techniques, such as nearest-neighbor matching. The objective of tracking is to obtain a model of the motion between frames of the visual features, usually obtained by minimizing an objective function, such as reprojection or photometric error functions.

Matching features detected in consecutive frames is usually achieved using Iterative Closest Point (ICP) [112], as in [113], where large polygonal shapes are tracked. Notably, Tedaldi et al. [114] and Kueng et al. [115] similarly perform tracking with ICP binary templates obtained with the Canny edge detection algorithm [116] centered around Harris corners. In contrast, in previous approaches, the model template is generated from predefined patterns, for example, complex-shaped objects tracked by gradient descent [117] or by multiple kernels, e.g., Gabor filters, feeding a Gaussian tracker [118]. Glover and Bartolozzi [119] use a particle filter to improve over the previous approach applying the Hough transform to track a fast-moving ball [120].

Zhu et al. [121] approach the data association problem with a probabilistic framework that jointly optimizes matching with feature displacements in an Expectation–Maximization scheme. Gehrig et al. [81] propose EKLT that resolves the data association challenge with a generative model to predict the future appearance of generic features. Hence, they use Maximum Likelihood Estimation (MLE) to optimize the warp parameters and brightness increment velocity. While most previous techniques operate on intermediate representations that accumulate events in a traditional frame format, HASTE [122] aims to track on an event-by-event basis. Hence, they revisit a previous tracker formulation [123] with an efficient evaluation of the alignment score function that determines the transition among a discretized space of hypothetical states. Event Clustering-based Detection and Tracking (eCDT) [124] solves detection and tracking simultaneously with a novel clustering method that separates event groups based on the neighboring polarity and spatiotemporal adjacency. Finally, Messikommer et al. [125] trained a neural network to predict displacements by employing a correlation layer.

### 4.2. Optical Flow

Optical flow [126] is a technique for estimating the motion of objects or scenes in a sequence of images or video frames based on the apparent movement of pixels between frames. Optical flow computes a dense vector field that represents the displacement of each pixel in the image over time. Also, it can be used to track objects or scenes by identifying regions of similar optical flow using clustering techniques, such as mean shift. The task of optical flow is closely related to feature tracking, differing in that it computes a displacement vector for every pixel in the input frame rather than sparse keypoints, regardless of the detection algorithm. However, due to incomplete information, e.g., the lack of knowledge of the scene geometry, ambiguity, noise, and occlusion, the problem is ill-defined and requires additional constraints. For example, brightness constancy assumptions [127] or a local smoothness prior [128] are usually applied.

Unlike image frames, events do not contain the same amount of information that can be extracted from observing the absolute brightness directly on an image plane. Hence, early methods, such as [79,129,130,131], start with testing optical flow reconstruction on the simple motion vector field created by a rotating black bar pattern, which triggers events on a continuous spiral in the x−y−t space. Benosman et al. [129] propose an algorithm based on the Lucas-Kanade [128] coarse-to-fine iterative approach by computing the partial derivative over a small neighborhood of events. Later, Benosman et al. [79] refined this approach with an alternative formulation that finds the flow as the slope of a plane fitted on a spatiotemporal region of the event stream. Brosch et al. [130] considers multiple issues with previous approaches, such as the numerical instability of the gradient approximation approach or plane fitting that requires either too small or too many events for robust estimation. Hence, they suggest a methodology that measures velocity as the response of a family of Gabor filters tuned to different velocities and directions by fitting their frequency sensitivity on the experimental data [132]. These early approaches have been compared on a common benchmark where the optical flow was generated from a camera rotating on its three axes, and an Inertial Measurement Unit (IMU) was used to generate ground truth from the gyro angular rates [133].

Similarly to the tuned Gabor filter, the SNN in [131] forms layers of neurons responding to eight speeds, eight directions, and on/off events on a 5 × 5-pixel region. The approach mimics the classic Lucas–Kanade in a bio-inspired framework. In contrast, Paredes-Valles et al. [134] demonstrate learning the neurons’ connection parameters from unsupervised data with a hierarchical SNN architecture. To this aim, they introduce a novel adaptive mechanism for the Leaky Integrate-and-Fire neurons and a stable implementation of the Spike-Timing-Dependent Plasticity (STDP) learning protocol. Additionally, they released the code for simulating a large SNN on GPU-accelerated hardware in an open-source library, cuSNN. More recent approaches, e.g., [135,136,137], have drastically improved the accuracy performance either by combining artificial neural networks for extending to deeper layers or by adopting more complex architectures [138].

Instead of computing flow on the raw events, Bardow et al. [139] propose jointly estimating the image log intensity with the velocity field in a sliding window variational optimization scheme. Besides demonstrating high dynamic range frame reconstruction, this approach can obtain a dense optical flow field. However, in areas where events have not been received, the optical flow is less reliable, as it results only from the constraints in the optimization equations, such as smoothing regularization terms.

Contrary to Lucas–Kanade-inspired works, Liu and Delbrück [54] propose a method based on block matching, a technique widely used for video compression. They extend their previous FPGA implementation [140] with more efficient computations for real-time operation. Remarkably, they accumulate 2D histograms of events in three adaptive time slices that are continuously rotated. Then, they found the best matching block of a region centered around an incoming event using the Sum of Absolute Difference (SAD) function. Subsequently, Liu and Delbruck [141] proposed a further improvement based on a novel corner detection algorithm implemented in hardware, SFAST, which allows for skipping computations for non-keypoint events.

Furthermore, deep learning has been applied to leverage the large availability of data. Due to the lack of ground-truth optical flow in the event domain, initial work approached the problem following a self-supervised learning paradigm [59,142,143]. They adopt models, e.g., U-Net, and loss functions from the standard camera deep learning research that can learn the 3D structure and the motion of the camera together with the flow. Moreover, the diverse nature of events requires finding the input representation that preserves the most information [144]. Hence, while exploring slight variations in the input format, recent methods introduce correlation cost volumes [145], recurrent units [146], and transformer blocks [147], usually in an encoder–decoder architecture fashion. More recently, BlinkSim [148], a simulator of actual event data and optical flow ground truth based on the Blender 3D engine, has been released, allowing further tuning of deep learning models.

### 4.3. Camera Localization and Mapping

Estimating a camera’s 6-DoF pose is fundamental to enabling autonomy in robotics and vision systems, underpinning tasks such as navigation, mapping, and interaction with the environment [93]. When both mapping and localization occur simultaneously in an unknown environment, the task is referred to as Simultaneous Localization and Mapping (SLAM) [149,150].

Although SLAM can rely on various sensors, including LiDAR, IMU, RADAR, or even radio-based methods [151], event cameras naturally align with visual SLAM (VSLAM). Their low latency, high temporal resolution, and robustness to motion blur and lighting changes make them attractive alternatives. However, the asynchronous data stream they produce challenges conventional SLAM pipelines, which typically assume a fixed frame input.

Event cameras output sparse, high-frequency brightness changes rather than global frames. As a result, SLAM algorithms must be restructured to handle this format, often using feature-based or direct methods. Many systems represent the scene with semi-dense edge maps co-estimated with camera pose, leveraging the fact that events are primarily triggered by edge motion [152,153,154].

Early event-based SLAM systems were predominantly feature-based, extracting and tracking corners or lines to estimate motion and build sparse 3D reconstructions [70,155,156]. Corner detectors such as eHarris [100], eFAST [101], and FA-Harris [105] were adapted to event data but often struggled with noise and motion variation. More recent methods improved stability by incorporating learning-based feature extractors, including recurrent networks and time-surface representations. Line-based tracking also added geometric constraints, and feature positions were typically optimized using probabilistic filters or bundle adjustment [82,157].

Direct approaches avoid explicit features and instead align event data with geometric or photometric models. Common strategies involve transforming events into image-like representations such as time surfaces and then aligning them against known scene structure or intensity maps [70,158]. Bayesian filtering is often used for incremental motion estimation, while methods like EVO [154] align event images with semi-dense maps. EMVS [159] introduced an efficient back-projection method to accumulate events in 3D space and recover depth from multiple viewpoints.

To improve performance in low-texture regions or during fast motion, many systems integrate IMU measurements. Visual–Inertial Odometry (VIO) pipelines such as Ultimate SLAM [160] or ESVIO [161] fuse event and inertial data, often using continuous-time trajectory models. Stereo event cameras have also been employed to recover depth through temporal and spatial consistency [162,163], while RGB-D setups like DEVO [164] combine event streams with depth sensors to enhance mapping fidelity.

Motion compensation remains key to improving spatial coherence. Techniques such as contrast maximization [152] or event-cloud alignment aim to sharpen accumulated events, supporting robust tracking even under fast motion or extreme lighting.

Loop closure and long-term consistency, while less explored, are gaining traction. Recent work applies spatiotemporal descriptors and graph-based optimization to reduce drift and improve global accuracy.

Deep learning has also become central to event-based SLAM. Early self-supervised approaches by Zhu et al. [59] and Ye et al. [143] showed that depth, optical flow, and ego-motion can be learned jointly from voxel-grid or time-surface representations. These models typically use CNN encoder–decoders trained with photometric or warping losses.

Subsequent work improved monocular depth estimation. Hidalgo-Carrio et al. [165] used recurrent CNNs to accumulate spatiotemporal information and predict dense depth from events alone. EMoDepth [166] refined this with a cross-modal training strategy: using aligned frames only during training while operating with events alone at inference, achieving state-of-the-art accuracy on MVSEC and DSEC.

Pose relocalization also benefited from deep models. CNN–LSTM networks [167] and transformer-based approaches like AECRN [168] exploit entropy-based event representations to regress 6-DoF pose. PEPNet [169] introduced a point-based model that processes raw event streams as 4D point clouds, outperforming prior work while remaining lightweight. Spiking neural networks (SNNs) have been explored for their potential efficiency on neuromorphic hardware. Spike-FlowNet [135] combined ANN and SNN layers for optical flow, while a fully spiking approach by Hagenaars et al. [170] achieved comparable results with much lower energy cost. Although many learned methods still rely on auxiliary frames or depth maps during training, the trend is moving toward fully event-driven models. Progress in spatiotemporal event representations—such as entropy frames, voxel grids, or point clouds—alongside attention modules, recurrent encoders, and spiking networks, is making real-time, frame-free SLAM increasingly feasible.

For evaluation, most approaches rely on public benchmarks such as MVSEC [171], DSEC [172], the IJRR Event Camera Dataset [173], and M3ED [174].

Event-based SLAM remains an evolving frontier. While feature-based and direct methods offer complementary strengths, major challenges persist in scalability, robustness, and fusion. Continued development of hybrid pipelines, neuromorphic hardware, and self-supervised learning is likely to drive future advances in autonomous event-based systems.

### 4.4. Moving Object Detection

Motion detection clearly highlights the advantages of neuromorphic photoreceptors compared to standard cameras. Thanks to their event-driven nature, neuromorphic sensors offer higher temporal resolution and faster responses, providing a more efficient way to detect moving objects. Unlike standard cameras, which rely on sequences of intensity frames and indirect measurements (such as optical flow), event cameras directly sense motion as changes occur in the scene. Under constant lighting and stationary camera conditions, segmenting moving objects becomes relatively straightforward, as only moving elements trigger events [175]. However, when the camera itself is moving, separating object motion from the camera’s ego-motion becomes more complex.

Initial efforts to tackle this challenge relied on classical computer vision techniques adapted to neuromorphic sensing. For instance, Glover and Bartolozzi [120] successfully tracked a fast-moving ball with an event camera mounted on the iCub robot by integrating Hough-transform circle detection with optical flow techniques, achieving robust detection at 500 Hz despite significant background clutter caused by robot movement. Similarly, Vasco et al. [176] leveraged the joint velocities of the robot to distinguish the motion of independent objects from the motion induced by the camera, effectively tracking the general shapes of objects.

To improve robustness under ego-motion, researchers explored motion-compensated representations of event data [69], which align events into sharp images by estimating and removing the camera’s motion. This approach enabled Mitrokhin et al. [84] to detect moving objects through motion inconsistencies and was further extended by Stoffregen et al. [177], who introduced a clustering method that jointly estimates object motions to refine segmentation results.

As deep learning entered the field, early models were adapted specifically for object detection using event data. Cannici et al. [178] proposed YOLE and fcYOLE, two neural architectures designed to process events either through integrated surfaces or in a fully asynchronous manner. These models demonstrated the feasibility of adapting frame-based convolutional techniques to sparse event streams. Building on these ideas, Liang et al. [179] introduced GFA-Net and CGFA-Net—transformer-based detectors evaluated on the EventKITTI dataset that combine local feature extraction with global context through edge-aware position encoding.

Expanding on these foundational approaches, Mitrokhin et al. [180] presented a more integrated neural network-based pipeline for motion segmentation. Their model simultaneously estimated depth, ego-motion, segmentation masks, and object velocities. They also introduced the EV-IMO dataset, providing detailed pixel-wise annotations in challenging indoor scenes. Later, the EVIMO2 dataset [181] expanded these benchmarks with greater complexity and more extensive annotations, facilitating robust training for both supervised and semi-supervised methods.

In parallel, neural architectures were refined to exploit the asynchronous nature of events better. For instance, spiking neural networks, previously explored for optical flow tasks, have also shown promise in segmentation. SpikeMS [138] applied a deep spiking encoder–decoder architecture to motion segmentation using DVS input, achieving performance comparable to artificial neural networks while significantly reducing energy consumption. Recent approaches, like the Recurrent Vision Transformer (RVT) by Gehrig and Scaramuzza [182], began employing transformer architectures to fully leverage event data’s temporal and spatial properties. The RVT reached state-of-the-art results on automotive detection benchmarks (Prophesee GEN1), achieving extremely low latency detection and demonstrating that transformer models could significantly enhance event-based object detection.

To address complex outdoor scenes where ego-motion plays a dominant role, methods like EmoFormer by Zhou et al. [183] have emerged. EmoFormer cleverly uses events only during training to inject strong motion awareness into a segmentation network, which then performs segmentation using only standard images at inference. They introduced the DSEC-MOS dataset, providing pixel-wise motion annotations for driving scenarios and addressing a critical gap in available training data. A complementary approach by Georgoulis et al. [184], called “Out of the Room”, explicitly compensates for ego-motion using monocular depth estimation before segmenting independently moving objects, further setting new benchmarks on EV-IMO and DSEC-MOTS datasets.

Given the difficulty and cost of labeling event data, recent methods also explored unsupervised or semi-supervised strategies. Un-EvMoSeg by Wang et al. [185] introduced an entirely unsupervised method using geometric constraints to detect independently moving objects without needing labeled data, achieving competitive results compared to supervised approaches. Similarly, LEOD, proposed by Wu et al. [186], uses pseudo-labels and temporal consistency to train detectors effectively with minimal supervision, demonstrating strong results with very few annotations.

Beyond neural networks, researchers have also drawn inspiration from biology. The retina-inspired Object Motion Sensitivity (OMS) framework, initially introduced by Snyder et al. [187] and further advanced into a low-overhead segmentation algorithm by Clerico et al. [188], emulates retinal circuits to discriminate object motion from ego-motion. This bio-inspired approach enables lightweight, efficient segmentation of moving objects directly from event-camera data, avoiding the need for explicit ego-motion compensation. Another non-learning approach, JSTR by Zhou et al. [189], combined IMU measurements and geometric reasoning to segment moving objects effectively, showcasing robust results without relying on heavy learning frameworks.

Hybrid methods combining event data with other modalities, particularly RGB frames, have also proved valuable. For instance, RENet [190] fuses event data and standard RGB images using attention mechanisms, greatly improving object detection accuracy under diverse conditions, including challenging lighting and rapid motion scenarios. Another notable hybrid approach, FlexEvent, introduced by Lu et al. [191], focuses on adapting object detection to arbitrary event frequencies. It combines event data with RGB frames using an adaptive fusion module (FlexFuser) and a frequency-adaptive learning strategy (FAL), achieving robust object detection performance across frequencies ranging from 20 Hz up to 180 Hz. This flexibility makes it particularly suitable for dynamic, real-world scenarios where event rates vary significantly.

As new datasets expand the range of evaluation scenarios, the field steadily bridges the gap between low-level motion cues and high-level scene understanding. With approaches ranging from fully event-based models to hybrid and unsupervised methods, current systems are increasingly capable of accurate, real-time segmentation, even in challenging, dynamic environments.

### 4.5. Spiking Neural Networks for Event-Based Processing

Spiking neural networks (SNNs) have emerged as an effective computational paradigm for processing the sparse, asynchronous data streams generated by neuromorphic sensors. Their spike-based coding schemes naturally exploit temporal dynamics and enable low-power computation, making them well suited for event-driven tasks. Recent advances have focused on scaling SNN architectures by incorporating design principles from deep learning, notably convolutional residual networks and transformers, to improve representational capacity, training stability, and hardware compatibility.

A core challenge in deep SNNs is preserving temporal features across many layers without experiencing vanishing or exploding gradients. Conventional deep CNNs solve this via residual connections, which protect important temporal information through the layers, enabling deeper network architectures that can efficiently process both spatial and temporal features in event-driven data. However, spiking analogs struggle due to the discrete, non-linear dynamics of spiking neurons. Fang et al. [192] addressed this with the Spike-Element-Wise (SEW) residual block, which adapts the identity mapping to spike-based signals through element-wise operations (ADD, AND, IAND) between the residual and shortcut paths. This design guarantees gradient flow across hundreds of layers, enabling direct training of ultra-deep SNNs (e.g., >100 layers) without degradation. Empirical results show that SEW-ResNet outperforms previous directly trained SNNs in both accuracy and temporal efficiency. Other CNN–SNN hybrids build on these ideas, combining spiking convolutions with temporal pooling or biologically inspired kernels to further enhance spatial–temporal feature extraction. The inclusion of residual connections has been shown to significantly improve the performance of spiking CNNs in tasks like dynamic scene recognition and object tracking [192].

While CNN-based SNNs excel at local spatial–temporal feature extraction, they lack global context modeling. Transformer [193] models have gained attention for their ability to capture long-range dependencies in event data. A significant challenge in transposing these models to event data is the design of spike-driven self-attention mechanisms that can effectively process sparse and asynchronous spike-based inputs, as conventional self-attention is ill-suited to sparse, asynchronous spikes. Recent works [194,195,196,197] have introduced novel spike-based approximations to attention, improving both efficiency and scalability. Yao et al. [194] proposed an efficient spike-driven self-attention mechanism that replaces multiplications with addition-only spike operations, making it hardware-friendly and energy-efficient. Their Spike Firing Approximation (SFA) training strategy bridges the representation gap between ANN and SNN attention layers, enabling large-scale training and competitive performance with ANN Vision Transformers on ImageNet-scale classification, detection, and segmentation. Related works refine spike-based attention for long-range temporal modeling in spikes. The Spiking Transformer [195] introduces an addition-only self-attention (A2OS2A) that removes softmax/scaling and mixes binary/ReLU/ternary units to reduce information loss while maintaining non-multiplicative computation, reaching 78.66% ImageNet-1k accuracy. Spatial–temporal attention [197] integrates time and space within spiking self-attention via block-wise processing at the same complexity, improving static and neuromorphic benchmarks. Hybrid Spiking Vision Transformer [196] targets event-camera detection by coupling ANN spatial modules with SNN temporal modules, showing gains on multiple benchmarks. Spatial–temporal spiking transformers can be inserted after spiking CNN stages to supply global context with bounded complexity, supporting the front-end/back-end split for event streams. Methods for conversion from artificial neural networks (ANNs) to SNNs have also seen significant progress. TTFSFormer [198] leverages Time-To-First-Spike (TTFS) coding to transfer pre-trained ANN transformers into the spiking domain with minimal accuracy loss, reducing the need for costly retraining. These advancements enable spiking transformers to achieve state-of-the-art performance in tasks such as event-driven object detection and sequence modeling by accurately capturing temporal dependencies in complex event streams.

SNN architectures have demonstrated strong results in a variety of event-driven vision tasks: from image segmentation, where Spike2Former [199] performs segmentation on event data, combining high spike efficiency with performance comparable to frame-based methods, to dynamic scene super-resolution, with examples such as Spk2SRImgNet [200], which addresses the problem by leveraging motion-aligned collaborative filtering techniques. Beyond these task-specific advances, the development of dedicated datasets further supports the evaluation and comparison of SNN-based methods. Datasets such as UCF-Crime-DVS [201] provide valuable benchmarks for anomaly detection, complementing established neuromorphic datasets (e.g., Shifted MNIST [178], DVS Gesture [202], N-Caltech101-DVS [203], CIFAR10-DVS [204]) for systematic model evaluation.

Despite these advances, key challenges remain: efficient large-scale training, improved interpretability, and the integration of CNN and transformer strengths into unified spiking architectures. However, the strengths of SNNs, particularly their temporal dynamics, sparsity-driven efficiency, and compatibility with neuromorphic hardware, position them as key enablers for future event-driven sensor technologies. Neuromorphic vision chips such as the Speck and Tianmouc [43,44] platforms underscore that algorithm–hardware co-design for spiking vision is feasible today and motivates SNN backbones tailored to such chips. Continued progress in spiking architectures, including hybrid models that combine the strengths of CNNs and transformers, and the development of more efficient training techniques will be crucial for unlocking the full potential of SNNs in real-time, event-based tasks.

## 5. Applications

**Figure 8:**
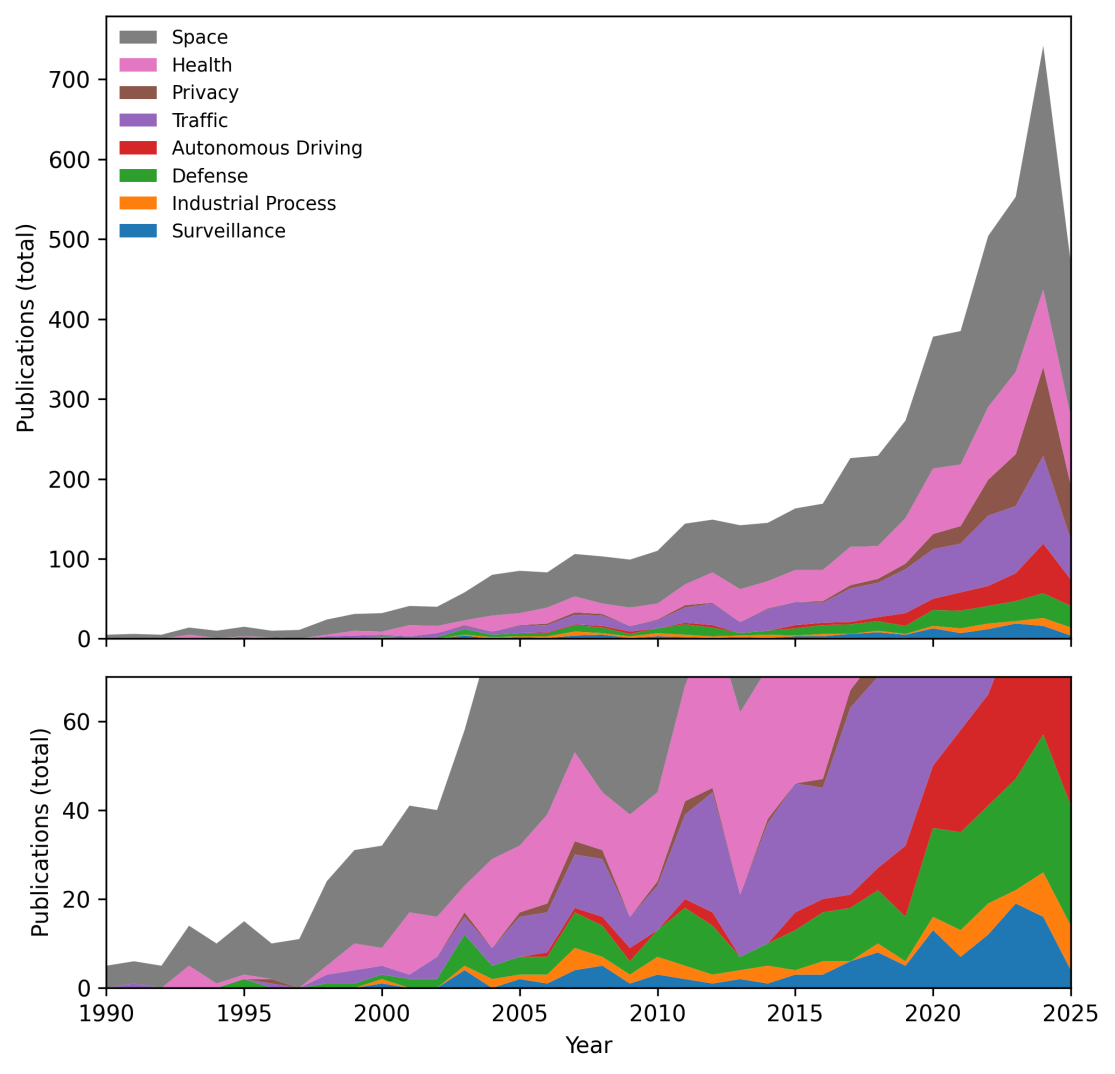
Application trends over time. The figure’s legend entries are ordered by descending number of publications in 2025.

This section discusses the various applications of event cameras across different domains, emphasizing their advantages and challenges in real-world settings. Figure 8 below shows the trend in applications over time, based on a search with specific queries in the *Dimensions.ai* database. It reflects the growth and diversification of event-camera applications in fields such as healthcare, autonomous driving, and surveillance.

The principal qualities of event cameras, namely, microsecond-level temporal resolution, low latency derived from independent pixel circuits, minimal power consumption (around 1 mW), reduced memory usage due to sparse output, and high dynamic range for visual sensing under sunlight or moonlight [205], align well with the functional needs of several domains. These include search and rescue, surveillance, autonomous driving [206], traffic monitoring [207], power line inspections [208], Industry 4.0, and situational awareness in space [209,210,211]. In these scenarios, event cameras enable robust vision despite motion, extreme lighting, or power constraints. For example, high temporal resolution is essential for fast-moving robotics; high dynamic range benefits outdoor and orbital observation; and low power consumption is key in mobile or embedded platforms. Augmented and Virtual Reality (AR/VR) applications, such as eye tracking and gesture recognition [212], benefit similarly from low latency and data sparsity. In Table 6, we associate specific sensor strengths with the application domains where they provide measurable technical benefits and advantages compared to classical vision systems based on insights from the literature review summarized herein.

### 5.1. Health and Sport-Activity Monitoring

Event cameras can capture detailed information about an individual’s activity, including detecting falls, tracking movements, and analyzing gait patterns, and could be applied to provide early warning signs of health issues or injuries. Several works have recently been proposed to estimate the human body pose from event-camera measurements, e.g., [161,213,214,215,216,217], a task for which a dedicated dataset has been released [218]. In healthcare, event cameras are being explored for applications such as surgical monitoring and neural imaging, where the ability to capture subtle, fast physiological motions can improve diagnostic accuracy [219,220]. Furthermore, combining high-temporal-resolution events with color images allows for interpolating new frames faster than the original video stream, reducing ghosting and other artifacts caused by non-linear motions [221,222].

### 5.2. Industrial Process Monitoring and Agriculture

Event cameras are emerging as powerful tools for industrial environments that demand high-speed, high-precision monitoring. Their low latency, high temporal resolution, and robustness to lighting variations make them particularly well suited for real-time quality control, equipment diagnostics, and predictive maintenance. In agriculture, their asynchronous operation and high temporal resolution open new avenues for real-time crop monitoring and precise field management, enhancing precision farming techniques [223].

One illustrative use case is high-speed object counting. For example, Bialik et al. [224] demonstrated a Prophesee EVK1 event camera successfully counting corn grains on a fast-moving feeder line, showcasing the potential of neuromorphic vision in manufacturing and logistics applications.

Beyond object counting, NCs have shown promise in broader industrial process monitoring tasks. For instance, Dold et al. [225] investigated the use of event cameras for laser welding, a domain where conventional photodiodes and high-speed cameras are typically used. Their study demonstrated that event cameras could visualize welding dynamics with superior temporal fidelity and detect production anomalies using learned representations.

In vibration monitoring, a critical task for predictive maintenance and structural diagnostics, Baldini et al. [226] used event cameras to track mechanical vibrations with an accuracy comparable to expensive laser Doppler vibrometers. Their system combined stereo event tracking and video reconstruction (via E2VID [227,228]) to measure subtle displacement patterns at sub-pixel resolution.

These examples reflect the increasing adoption of event cameras for industrial process monitoring, which requires high-frequency observation and fast decision-making from precision manufacturing to large-scale industrial systems.

### 5.3. Space Sector

Neuromorphic sensors can be applied to telescopes to track stars [209], satellites [229], or debris in orbit [230] from the ground to avoid potential damage to other infrastructures. Recent research suggests that NCs with high spatial and temporal resolution may be exploited to identify the material of satellites [231]. Furthermore, Jawaid et al. [232] leverage the high dynamic range of the event sensor to estimate satellite pose to ensure robustness to drastic illumination changes. Also, Mahlknecht et al. [233] demonstrate that event cameras are suitable for planetary explorations where challenging scenarios, such as the Mars landscape, pose many challenges in estimating the autonomous robot self-position. The International Space Station (ISS) has an event-based sensor to detect lightning and sprite events in the mesosphere. These events can occur in as little as 100 microseconds [234].

### 5.4. Surveillance and Search and Rescue

NCs are well suited for monitoring public spaces or securing buildings due to their low power consumption and real-time processing capabilities. These features enable them to monitor large areas without frequent maintenance or battery replacements. Furthermore, traditional surveillance cameras can be limited in accurately detecting and tracking objects in complex and dynamic environments where the HDR capabilities of these sensors compensate for lighting variability factors and nighttime operations. Research has shown that NCs can detect and track multiple moving objects in real time, even if many challenges of complex environments must still be addressed [235]. Ganan et al. [236] propose an event-based processing scheme for efficient intrusion detection and tracking of people using a probabilistic distribution and CNN, which has been validated in various scenarios on a DJI F450 drone.

Aerial robots are the primary platform for surveillance and search and rescue applications. Mainly, event-based cameras’ high temporal resolution and dynamic range help handle the motion blur caused by UAVs while detecting and tracking possible intruders [237]. Recent work by Rodriguez-Gomez et al. [238] introduces an asynchronous event-based clustering and tracking method for intrusion monitoring in a UAS. Their approach leverages efficient event clustering and feature tracking while incorporating a sampling mechanism to adapt to hardware constraints, demonstrating improved accuracy and robustness in real-world scenarios. Deep learning methods for event-based human intrusion detection in UAV surveillance have also been explored to gain more confidence in determining the type of moving object. Pérez-Cutino et al. [239] present a fully event-based processing scheme that detects intrusions as clusters of events and classifies them using a CNN to determine whether they correspond to a person. In particular, this method eliminates the need for additional onboard sensors and fully exploits the asynchronous nature of event cameras.

Similarly to surveillance, event cameras are useful in search and rescue operations, especially in environments like forests or mountains, because of their high temporal resolution and low power consumption, allowing them to capture detailed information about the environment and providing real-time feedback to rescuers while moving fast and for extended missions. More importantly, the low latency of NCs can allow remote UAV pilots to perform more aggressive flights [240], which is critical to reducing operation time while safely avoiding obstacles in unknown cluttered environments [241].

### 5.5. Autonomous Driving

Autonomous driving applications like collision avoidance could benefit from event cameras. With their low latency and high temporal resolution, they can capture detailed information about the environment and provide real-time feedback to the autonomous driving system. Hence, they will be an essential resource for implementing advanced driver assistance systems (ADASs) and self-driving cars in the future. To this aim, Wzorek and Kryjak [242] recently demonstrated how a neural network could detect traffic signs. Not only that, but event cameras have also been tested on the driver distraction detection task by Yang et al. [243], where the authors evaluated the proposed approach by converting standard video clips with an event simulation tool [244].

### 5.6. Traffic Monitoring

Event cameras may be helpful for traffic monitoring applications, such as estimating car speed [207]. Their low power consumption and real-time processing capabilities make them well suited for monitoring large areas without frequent maintenance or battery replacements. Therefore, event cameras can detect and track multiple cars on the road simultaneously [245] or pedestrians and cyclists [246].

### 5.7. Defense

Event cameras offer significant advantages for defense applications due to their low power consumption, high temporal resolution, and ultra-low latency. These features make neuromorphic cameras ideal for embedded systems in UAVs and other autonomous platforms, enhancing obstacle detection, target tracking, and surveillance while maintaining power efficiency. In reconnaissance and battlefield monitoring, NCs provide continuous high-speed data streams that improve situational awareness in real time [247]. Their ability to track fast-moving targets is particularly valuable for Unmanned Ground and Underwater Vehicles (UGVs and UUVs), where reaction time is critical [248].

Recent studies have also explored using NCs for laser warning and detect-before-launch (DBL) capabilities. For instance, Boehrer et al. [249] demonstrate how the high temporal resolution of event cameras can be leveraged to detect laser emissions and retro-reflections from pointed optics, key indicators of hostile intent. Their system was evaluated in operational scenarios during the DEBELA trial, showing that event-based sensing enables early and reliable threat detection. Complementing this work, the DEBELA project [250] investigates electro-optical technologies for future self-protection systems, focusing on within-visual-range missile threats that are difficult to detect using conventional sensors.

NCs also show promise in Counter-Unmanned Aerial Systems (C-UASs), where their ability to capture fast-moving drones or hypersonic missiles can aid early warning systems. Their sensitivity in the infrared and short-wave infrared bands allows for enhanced night vision and detection of low-signature propellants [251,252]. Together, these capabilities position event-based sensors as powerful tools for modern defense, offering real-time threat perception, reduced false alarms, and greater autonomy in decision-making.

### 5.8. Other Emerging Applications

The range of industries exploiting neuromorphic cameras is unlimited. The last years have produced a handful of proof-of-concept studies that push neuromorphic vision into domains largely absent from the mainstream literature. We outline three particularly promising, yet still underexplored, directions that complement the existing catalog of applications of the survey.

Low latency and high dynamic range permit detection of the subtle chromatic and geometric changes caused by blood flow or respiration. A recent proof-of-concept study showed accurate heart rate estimation from facial events while using dramatically less data and power than frame-based approaches [253]. Public multi-subject datasets and standard evaluation protocols are still missing, making this a ripe area for first-mover contributions.

Because they record only brightness changes, event cameras discard most static texture information that fuels face or scene re-identification. Indoor-localization prototype algorithms have demonstrated that event-only maps can enable accurate positioning while transmitting almost no personally identifiable visual content [254]. The next step is to formalize threat models and privacy metrics so that lightweight encryption and anonymization schemes can be co-designed with resource-constrained inference.

Ultra-low-power spiking neural network chips are now being paired directly with event sensors in sense–process–act stacks that run entirely on the edge. Field trials in battery-powered drones and smart home nodes report faster response times and substantial energy savings compared to CNN frame-based baselines [255]. Open challenges remain in tailoring event representations to tiny on-chip memories and scheduling heterogeneous cores under single-digit milliwatt power caps.

Finally, neuromorphic vision systems can be integrated into sensor fusion frameworks, combining modalities such as inertial sensors, microphones, or bio-signals to enhance situational awareness. For instance, Kiselev et al. [256] demonstrate a real-time FPGA-based system combining a DVS with a Dynamic Audio Sensor (DAS), achieving significantly higher classification accuracy through multi-modal input. Similarly, O’Connor et al. [257] present a spiking Deep Belief Network that fuses input from a silicon retina and cochlea, achieving robust performance even under sensory noise. These examples highlight how event-based fusion can enrich perceptual systems in fields like mobile robotics, smart wearables, and embedded AI.

## 6. Discussion

In this section, we delve deeper into the identified gaps in the current stage of development in the field of neuromorphic vision, drawing insights into the directions that research and industry could take to capitalize on the multiple opportunities this sensor offers. Hence, in Table 7, we provide an overview of the gaps and future directions that we analyze in more detail herewith.

### 6.1. Gap Analysis

Despite considerable advancements in neuromorphic sensors and algorithms, several gaps remain that prevent their widespread adoption and limit their ability to fully replace classical vision sensors.

At the hardware level, the primary limitations are sensor availability, manufacturing complexity, and cost. Neuromorphic sensors remain expensive due to their specialized manufacturing processes, restricting broad commercial availability. Additionally, current event cameras typically provide lower spatial resolution compared to traditional frame-based sensors, limiting their effectiveness in applications demanding high detail. Another significant hardware constraint is the limited spectral range, with most sensors operating only in the visible spectrum. Although early initiatives like the DARPA FENCE program and recent developments of infrared-sensitive neuromorphic sensors (e.g., SWIR-sensitive cameras) exist, these efforts are still at an early stage, limiting widespread implementation.

Algorithmically, a major challenge arises from fundamental differences between event-based and conventional visual data. Event-based vision algorithms are comparatively less mature and require new data representation methods and processing approaches. Although methods like voxel grids, time surfaces, and event histograms have emerged, a universally accepted approach adaptable across multiple vision tasks is still lacking. The continuous and asynchronous nature of sparse event streams poses significant challenges to developing robust algorithms and represents a substantial paradigm shift from traditional computer vision techniques. Notwithstanding their sparse nature, real-time processing and intelligent clustering of event streams remain challenging, as managing the high volume of events and extracting relevant information is nontrivial. Additionally, benchmarks and standardized evaluation frameworks designed explicitly for event-based data remain limited, impeding progress in algorithm validation.

At the application level, neuromorphic vision systems are primarily limited to laboratory prototypes, with few robust, commercially viable solutions available. Achieving consistent performance in uncontrolled, dynamic environments remains difficult; particularly significant challenges arise from environmental noise such as intermittent lighting variations and sensor-induced noise, requiring more advanced noise filtering methods. For example, critical tasks such as event-based visual SLAM, which is paramount for the future of autonomous driving or other robotic contexts, still struggle with drift reduction, effective loop closure detection, and reliable operation in complex real-world scenarios involving rapid movements or significant scene aspect changes.

Finally, fully exploiting the inherent energy efficiency advantages of neuromorphic sensors in practical deployments demands integration with specialized neuromorphic computing hardware, optimized explicitly for processing sparse and asynchronous event data. Current general-purpose hardware, such as CPUs and GPUs, lacks the efficiency for event-based processing, while dedicated neuromorphic computing platforms that support SNNs remain limited in commercial availability. Achieving widespread industrial use will require concerted efforts toward hardware innovation and software maturity, a challenge most companies are currently unable to tackle without greater standardization and market maturity.

### 6.2. Opportunities and Future Directions

However, despite the gaps discussed, several promising opportunities exist for further advancing neuromorphic vision technology.

In hardware, key opportunities include reducing sensor manufacturing costs through mass production and strategic industrial collaborations. Recent partnerships, such as that between Prophesee and Qualcomm, which aim at integrating event-based cameras into smartphones, and Google’s integration of neuromorphic sensors into Android XR for augmented reality, are paving the way for broader market adoption. Furthermore, neuromorphic chips like SynSense Speck, designed for ultra-low-power and high-speed imaging, can potentially extend event-based sensing to consumer electronics and affordable machine vision solutions. Expanding into infrared and non-visible spectral domains also presents significant potential, particularly for security, defense, and environmental monitoring applications. Additionally, integrating neuromorphic sensors into edge devices paired with neuromorphic processors, which significantly reduce power consumption and enhance real-time processing capabilities, presents another critical opportunity for practical implementations, especially in energy-constrained environments.

On the event-processing side, improving temporal neural networks remains important, including architectures like spiking neural networks (SNNs) and Long Short-Term Memory (LSTM), which naturally handle time-based event data. A growing body of work also explores learned event-based representations, which encode spatiotemporal patterns in formats better suited to downstream processing, signaling space for improvement in this area. Moreover, transformer-based models, initially developed for language processing [193] and later adapted to traditional computer vision [258], are starting to show potential for event-based vision tasks such as object detection, video reconstruction, and pose estimation. These models effectively capture long-term temporal dependencies in event data, offering advantages over conventional convolutional networks. In this context, sparse-aware transformer designs like the Event Transformer (EvT) [259] further improve computational efficiency by leveraging the unique sparsity of event streams, making them more suitable for real-time, resource-constrained applications.

Developing standardized deep learning benchmarks and datasets specifically for event-based vision tasks is critical to accelerating algorithmic maturity and adoption [15]. Advances in synthetic event-data generation tools (e.g., v2e [244]) that accurately emulate sensor behavior under varying conditions also offer significant potential to facilitate algorithm development and training, reducing dependency on extensive real-world data collection. These tools can further enhance algorithm robustness to environmental factors, such as noise, varying illumination, and complex scenes, by providing an extensive and controllable source of training data for neural network-based methods. Lastly, developing computationally efficient algorithms optimized for specialized neuromorphic hardware accelerators remains essential for enabling practical and widespread adoption.

Regarding applications, moving from laboratory prototypes to real-world industrial solutions remains a significant opportunity. Integrating neuromorphic sensors with traditional cameras and other sensor types (such as IMUs, LiDAR, and microphones) can combine strengths and significantly enhance system performance. In particular, event-based SLAM systems that leverage both neuromorphic sensing and neuromorphic computing represent an immediate opportunity, especially in complex environments where conventional sensors struggle, such as autonomous vehicles navigating dynamic urban settings, drones operating under variable lighting conditions, or robotic systems employed in search and rescue and defense applications.

Furthermore, several less explored application domains could notably benefit from neuromorphic sensors, opening new opportunities for adoption. For instance, agriculture and precision farming can leverage event-based vision, e.g., for real-time crop monitoring. Healthcare applications, particularly surgical assistance, patient monitoring, or even microexpression analysis for telemedicine, could exploit the sensitivity of neuromorphic sensors to rapid and subtle physiological changes. Additionally, applications in sports analytics, such as real-time ball tracking or athlete movement analysis, present another promising use case, given the sensor’s ability to precisely track high-speed objects without motion blur. Even in heavy industry, where traditional high-speed cameras are already used for equipment inspection and wear monitoring [260], a transition to event-based vision could improve temporal resolution and data efficiency under harsh, dynamic conditions.

Increased awareness and dissemination efforts are crucial to facilitating industrial adoption. Initiatives like the 4th International Workshop on Event-based Vision at CVPR 2025 and the NeVi 2024 Workshop at ECCV 2024 are already helping to connect academia and industry by highlighting practical benefits and driving interest in neuromorphic sensors. Similarly, industry-focused events such as the VISION Fair provide valuable opportunities to reach broader industrial stakeholders. Expanding participation in these events, supported by targeted promotional activities and strategic partnerships, will further encourage market adoption and raise industry awareness of event-based vision technologies.

These advances across event-driven hardware, algorithmic innovation, and industrial collaboration underscore the rapid progression and diversification of neuromorphic vision technologies. To contextualize these developments further, the next section synthesizes broader insights and key perspectives highlighted in recent comprehensive reviews [6,17].

### 6.3. Perspectives on Neuromorphic Computing

Recent comprehensive reviews emphasize that neuromorphic computing is entering a pivotal new phase, driven by cross-disciplinary advancements in hardware, algorithms, and practical deployments [6,17]. Unlike traditional von Neumann architectures, neuromorphic systems physically integrate memory and computation, exploiting event-driven operations, local plasticity, and extreme parallelism to achieve ultra-low-power and real-time performance. These characteristics uniquely position neuromorphic platforms as ideal solutions for edge computing, robotics, distributed sensor networks, and resource-constrained, time-critical applications.

Two complementary trajectories characterize the current evolution of the field. First, neuromorphic hardware is rapidly scaling in both neuron count and functional specialization. Crucially, a prominent trend identified in the latest literature is the tight integration of sensors and computation on single devices, enabling ultra-efficient, event-driven inference directly at the data capture point. Recent implementations, such as the Speck chip [43]—integrating an event sensor with a fully asynchronous spike-based processor—and the biologically inspired dual-pathway Tianmouc chip [44], exemplify this convergence, providing practical demonstrations of real-time, energy-efficient perception in challenging dynamic environments. Second, advances in specialized spiking neural network (SNN) algorithms complement these hardware innovations. Modern SNN architectures, particularly transformer-inspired models and sparse, event-driven learning approaches, are now significantly better aligned with emerging neuromorphic hardware capabilities [6,17]. These algorithmic innovations take advantage of the strengths of sensor-integrated hardware to address real-world challenges such as sparse data management, temporal misalignment, and efficient real-time processing.

However, the community underscores the critical need for tighter hardware–algorithm co-design, standardized benchmarking datasets, and accessible software toolchains. Open questions remain regarding the seamless integration of neuromorphic systems with conventional computing environments, scalable software ecosystems, and tangible demonstrations of application-level advantages compared to traditional AI solutions. The convergence of integrated neuromorphic hardware and sophisticated SNN algorithms represents a defining theme for the coming decade. We expect substantial advancements in adaptive, robust, and energy-efficient applications driven by efficient event-based biologically inspired architectures.

## 7. Summary

Neuromorphic vision sensors, or event-based cameras, represent a fundamental shift from traditional frame-based imaging. Unlike conventional cameras that record full images at fixed intervals, neuromorphic cameras capture visual information asynchronously by registering changes in brightness at each pixel. This results in sparse, low-latency, and highly efficient data regarding power consumption and storage. In addition, neuromorphic sensors have exceptionally high temporal resolution and dynamic range, allowing them to operate effectively in challenging lighting conditions and rapidly changing environments.

This review systematically covered the key dimensions of neuromorphic vision technology: the evolution of sensor hardware, the specialized algorithms developed to process event-based data, and their diverse applications. Hardware advancements highlighted include sensor architectures that evolved from early silicon-retina concepts to increasingly sophisticated designs that capture richer visual information, including colors and absolute light intensity, at higher resolution. Algorithmically, event-based processing has adapted and extended classical image-processing tasks, e.g., feature detection, optical flow estimation, visual odometry, and object tracking, to handle asynchronous event streams efficiently. Finally, neuromorphic cameras have demonstrated substantial potential in various practical fields, including robotics, autonomous vehicles, industrial automation, and surveillance, taking advantage of their unique capabilities to enhance real-time responsiveness and robustness to environmental dynamics.

## Figures and Tables

**Table 1:** Overview of previous surveys on neuromorphic vision (Part 1).

Reference	Year	Focus Topic	Key Highlights
This survey	2025	Comprehensive Overview (Hardware, Algorithms, Applications)	Provides a structured and up-to-date survey of neuromorphic vision, covering the evolution of hardware, key developments in event-based image processing, practical application case studies, and current limitations. Highlights challenges and opportunities for broader adoption across domains.
Kudithipudi et al. [6]	2025	Neuromorphic Computing at Scale	Proposes a forward-looking framework for scaling neuromorphic systems from lab prototypes to real-world deployments. Offers high-level guidance on architecture, tools, ecosystem needs, and application potential. It is especially relevant for stakeholders aiming at system-level integration.
Ghosh and Gallego [7]	2025	Event-based Stereo Depth Estimation	Extensive survey of stereo depth estimation with event cameras, encompassing both model-based and deep learning techniques. Compares instantaneous vs. long-term methods, highlights the role of neuromorphic hardware and SNNs for stereo, benchmarks methods and datasets, and proposes future directions for real-world deployment of event-based depth estimation.
Adra et al. [8]	2025	Human-Centered Event-Based Applications	Provides the first comprehensive survey unifying event-based vision applications for body and face analysis. Discusses challenges, opportunities, and less explored topics such as event compression and simulation frameworks.
AliAkbarpour et al. [9]	2024	Emerging Trends and Applications	Reviews a wide spectrum of emerging event-based processing techniques and niche applications (e.g., SSA, RS correction, SR, robotics). Focuses on technical breadth and dataset/tool support, with limited emphasis on foundational hardware or unified algorithmic frameworks.
Shariff et al. [10]	2024	Automotive Sensing (In-Cabin and Out-of-Cabin)	Presents a comprehensive review of event cameras for automotive sensing, covering both in-cabin (driver/passenger monitoring) and out-of-cabin (object detection, SLAM, obstacle avoidance). Details hardware architecture, data processing, datasets, noise filtering, sensor fusion, and transformer-based approaches.
Cazzato and Bono [11]	2024	Application-Driven Event-Based Vision	Reviews event-based neuromorphic vision sensors from an application perspective. Categorizes computer vision problems by field and discusses each application area’s key challenges, major achievements, and unique characteristics.
Chakravarthi et al. [12]	2024	Event Camera Innovations	Traces the evolution of event cameras, comparing them with traditional sensors. Reviews technological milestones, major camera models, datasets, and simulators while consolidating research resources for further innovation.
Tenzin et al. [13]	2024	Event-Based VSLAM and Neuromorphic Computing	Surveys the integration of event cameras and neuromorphic processors into VSLAM systems. Discusses feature extraction, motion estimation, and map reconstruction while highlighting energy efficiency, robustness, and real-time performance improvements.
Becattini et al. [14]	2024	Face Analysis	Examines novel applications such as expression and emotion recognition, face detection, identity verification, and gaze tracking for AR/VR, areas not previously covered by event cameras surveys. The paper emphasizes the significant gap in standardized datasets and benchmarks, stressing the importance of using real data over simulations.
Zheng et al. [15]	2023	Deep Learning Approaches	Extensively surveys deep learning approaches for event-based vision, focusing on advancements in data representation and processing techniques. It systematically categorizes and evaluates methods across multiple computer vision topics. The paper discusses the unique advantages of event cameras, particularly under challenging conditions, and suggests future directions for integrating deep learning to exploit these benefits further.

**Table 2:** Overview of previous surveys on neuromorphic vision (Part 2).

Reference	Year	Focus Topic	Key Highlights
Huang et al. [16]	2023	Self-Localization and Mapping	Discusses various event-based vSLAM methods, including feature-based, direct, motion-compensation, and deep learning approaches. Evaluates these methods on different benchmarks, underscoring their unique properties and advantages with respect to one another. Then, it gives deep reasons for the challenges inherent to sensors and the task of SLAM, drawing future directions for research.
Schuman et al. [17]	2022	Neuromorphic Algorithmic Directions	Highlights algorithmic opportunities for neuromorphic computing, covering SNN training methods, non-ML models, and application potential. Emphasis is placed on open challenges and co-design needs rather than exhaustive architectural or vision-specific coverage.
Shi et al. [18]	2022	Motion and Depth Estimation for Indoor Positioning	Reviews notable techniques for ego-motion estimation, tracking, and depth estimation utilizing event-based sensing. Then, it suggests further research directions for real-world applications to indoor positioning.
Furmonas et al. [19]	2022	Depth Estimation Techniques	Discusses various depth estimation approaches, including monocular and stereo methods, detailing the strengths and challenges of each. It advocates integrating these sensors with neuromorphic computing platforms to enhance depth perception accuracy and processing efficiency.
Cho et al. [20]	2022	Material Innovations and Computing Paradigms	Highlights the evolution from traditional designs to innovative in-sensor and near-sensor computing that optimizes processing speed and energy efficiency. It addresses the challenge of complex manufacturing processes, suggesting directions for future research and application in flexible electronics.
Liao et al. [21]	2021	Technologies and Biological Principles	Reviews advancements in neuromorphic vision sensors, contrasting silicon-based CMOS technologies such as DVS, DAVIS, and ATIS with emerging technologies in analogical devices.
Gallego et al. [3]	2020	Sensor Working Principle and Vision Algorithms	Thoroughly reviews the advancements in event-based vision, emphasizing its unique properties. The survey spans various vision tasks, including feature detection, optical flow, and object recognition, and discusses innovative processing techniques. It also outlines significant challenges and future opportunities in this rapidly evolving field.
Steffen et al. [22]	2019	Stereo Vision and Sensor Principles	Performs a comparative analysis of event-based sensors, focusing on technologies such as DVS, DAVIS, and ATIS. It reviews the biological principles underlying depth perception and explores the approaches to stereoscopy using event-based sensors.
Lakshmi et al. [23]	2019	Object Motion and SLAM	Reviews state-of-the-art event-based vision algorithms for object detection/recognition, object tracking, localization, and mapping. Highlights the necessity of adapting conventional vision algorithms. Also provides an overview of publicly available event datasets and their applications.
Vanarse et al. [24]	2016	Neuromorphic Vision, Auditory, and Olfactory Sensors	Highlights low power consumption in the prototypical developments of DVS and DAVIS using asynchronous spiking output. Suggests future research directions in neuro-biological emulating sensors for vision, audition, and olfaction with multi-sensor integration.

**Table 3:** Comparison of currently commercially available event cameras. Meps is millions of events per second.

Manufacturer	Model	Resolution	Latency	Temporal Resolution	Max Throughput	Dynamic Range	Power	Image Frames
IniVation	DAVIS346 (also, Color)	346 × 260	<1 ms	1 μs	12 Meps	120 dB	<180 mA	Graysc./Color
IniVation	DVXplorer	640 × 480	<1 ms	65–200 μs	165 Meps	90–110 dB	<140 mA	No
IniVation	DVXplorer Lite	320 × 320	<1 ms	65–200 μs	100 Meps	90–110 dB	<140 mA	No
IniVation	DVXplorer Micro	640 × 480	<1 ms	65–200 μs	450 Meps	90–110 dB	<140 mA	No
Prophesee	Gen 3 VGA CD	640 × 480	40–200 μs	NA	66 Meps	>120 dB	NA	No
Prophesee	GENX320	320 × 320	<150 μs	1 μs	NA	>120 dB	>36 μW	No
Sony/Prophesee	IMX636	1280 × 720	100–220 μs	NA	1060 Meps	86 dB	NA	Grayscale
Sony/Prophesee	IMX637	640 × 512	100–220 μs	NA	1060 Meps	86 dB	NA	Grayscale
Sony/Prophesee	IMX646	1280 × 720	800–9000 μs	NA	1060 Meps	110 dB	NA	Grayscale
Sony/Prophesee	IMX647	640 × 512	800–9000 μs	NA	1060 Meps	110 dB	NA	Grayscale
Imago Tech./ Prophesee	Vision Cam EB	640 × 480	200 μs	NA	30 Meps	>120 dB	NA	No
IDS/Sony/ Prophesee	uEye EVS	1280 × 720	<100 μs	<100 μs		>120 dB	10 μW	No

**Table 4:** Comparison of infrared neuromorphic cameras by wavelength.

Category	Wavelength	Capability	Applications
Short-wave (SWIR)	[1 μm–2.5 μ]	Organic vs. inorganic	Recycling, food, agriculture, and military
Mid-wave (MWIR)	[3 μm–5 μ]	Thermal radiation	Surveillance, thermography, and gas detection
Long-wave (LWIR)	[8 μm–14 μ]	Thermal radiation and ambient temperature	Imaging, night vision, and medical diagnosis

**Table 5:** Comparative analysis of event representations/preprocessing methods.

Method Family	Preserved Properties	What is Lost	Typical Tasks	Limitations	Representative References
**Event-by-Event Processing**	Max. temporal precision, polarity, sparsity	Local spatial context (unless modeled)	Low-latency tracking, control, VO, gesture	High per-event cost, poor GPU batching	[33,52]
**Temporal Binary Representation (TBR)**	On/off activity, memory efficiency, sparsity	Counts, time precision	Gesture/activity recognition	Severe info loss for regression tasks	[53]
**Event Frames**	Spatial context, polarity	Microsecond timing, sparsity	Optical flow, stereo, CNN steering	Motion blur for long windows	[54,55]
**Time Surfaces (SAE, TS, SITS)**	Newest timestamp per pixel, sparsity	Older timestamps	Corner detection, vSLAM	Normalization tuning; slow/ low-light degradation	[56,57,58]
**Voxel Grids**	Polarity, temporal bins	Intra-bin timing	DL pipelines, flow, depth	Quantization, memory grows with bins	[59,60,61]
**TORE Volumes/** **Temporal Queues**	*K*-recent timestamps/pixel	Older than *K*	Denoising, classification	Extra memory/ bandwidth for *K*	[62]
**Graph/** **Event-Cloud Encodings**	Full sparsity, exact timestamps, polarity; local topology	Dense-grid regularity (less GPU-friendly)	GNN-based classification	Graph construction cost/fixed size	[63,64]
**Learned Grids (ESTs, Matrix-LSTM, NEST, GWD)**	Task-optimized mix of timing, polarity, spatial context	Some interpretability, ultra-low latency	SOTA detection, reconstruction, SR, deblurring	Needs data, more compute	[60,61,65,66,67]

**Table 6:** Event-camera applications by domain vs. strengths leveraged. The checkmarks represent sensor capabilities that are technically beneficial to each application.

Application Domain	High Temp. Res.	Low Latency	High Dyn. Range	Low Power	Data Sparsity	Motion Robustness
Health and Sport Monitoring	✓			✓		✓
Industrial Monitoring	✓	✓	✓	✓	✓	✓
Space Sector	✓		✓	✓		✓
Surveillance and Rescue	✓	✓	✓	✓	✓	✓
Autonomous Driving	✓	✓	✓		✓	✓
Traffic Monitoring		✓		✓		
Defense	✓	✓	✓	✓	✓	✓
Others	✓			✓	✓	

**Table 7:** Gap analysis and future directions overview.

Level	Gap Analysis	Opportunities and Future Directions
	Sensor availability	Lower manufacturing costs, mass production
	Manufacturing complexity	Industrial collaborations (e.g., Prophesee)
**Hardware**	High sensor cost	Infrared, non-visible spectrum
	Low spatial resolution	Neuromorphic chips for consumer electronics
	Limited spectral range	Edge devices, low power, real-time processing
	Immature event-based algorithms	Improve SNNs, LSTMs
	Lack of universal data representation	Neural Event Stacks (NEST)
**Algorithmic**	Real-time processing challenges	Transformer models (object detection, etc.)
	Lack of benchmarks	Standardized event-based benchmarks
	Sparse data management	Synthetic event-data generation (e.g., v2e)
	Lab prototypes	Real-world industrial solutions
	Poor performance in dynamic environments	Integration with traditional sensors
**Applications**	Poor event-based SLAM performance	Event-based SLAM in complex environments
	Limited commercial solutions	Autonomous driving, robotics, surveillance
	Drift, loop closure issues	Low latency, high temporal resolution

## Data Availability

No new data were created or analyzed in this study. Data sharing is not applicable to this article.
